# Supplementary material for: SYCP2 recruits HORMAD2 to chromosome axes for unsynapsed chromatin silencing and synapsis surveillance in meiosis
Source: Nat Commun. 2026 Jul 23;17:7122. doi: 10.1038/s41467-026-75839-3 (PMC13396342; doi:10.1038/s41467-026-75839-3)
Supplement: Supplementary file 1 — Supplementary Information [file 41467_2026_75839_MOESM1_ESM.pdf]

SUPPLEMENTARY INFORMATION

SYCP2 recruits HORMAD2 to chromosome axes for unsynapsed chromatin silencing and synapsis surveillance in meiosis

Author list:

Kavya Raveendran§ [1], Sarai Valerio-Cabrera§ [1], Arkasarathi Gope[1], Vladyslav Telychko[1], Geen George [1], Christin Richter [1], Tanja Scholte [1], Matthias Weigel [1], Anastasiia Bondarieva [1], Andreas Petzold [2], Andreas Dahl [2], Kevin D Corbett [3,4], Attila Tóth [1@]

Affiliations

[1] Institute of Physiological Chemistry, Faculty of Medicine at the TU Dresden, Fiedlerstrasse 42 01307 Dresden, Germany

[2] DRESDEN-concept Genome Center (DcGC), Center for Molecular and Cellular Bioengineering (CMCB) Technology Platform, TUD Dresden University of Technology, 01062 Dresden, Germany

[3]Department of Cellular and Molecular Medicine, University of California, San Diego, La Jolla, CA, USA.

[4]Department of Molecular Biology, University of California, San Diego, La Jolla, CA, USA.

§ Equal contribution

[@]Correspondence to attila.toth@mailbox.tu-dresden.de

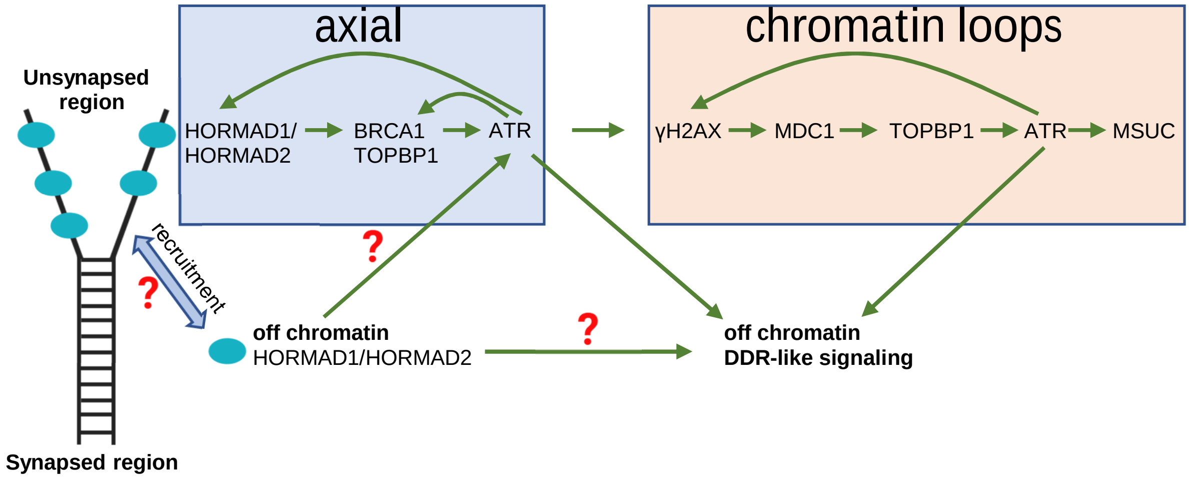

Supplementary Figure 1. Current model and outstanding questions regarding the molecular network underlying asynaptic axis–triggered ATR activation and the resultant prophase checkpoint in mammalian meiosis.

Green arrows indicate promoting effects; the blue double-headed arrow denotes inferred but undefined physical interactions that enable recruitment of HORMAD1 and HORMAD2 to unsynapsed axes. The prevailing model of the synapsis checkpoint proposes that preferential enrichment of HORMAD1 and HORMAD2 on unsynapsed chromosome axes forms the basis of asynapsis sensing 1–7. Axis-bound HORMADs are thought to establish an ATR-mediated signalling cascade that is reinforced by positive feedback and feedforward mechanisms 8–18, resulting in robust ATR activity on both axes and associated chromatin loops within unsynapsed regions. This signalling drives meiotic silencing of unsynapsed chromatin (MSUC) and DNA damage response (DDR)–like signalling, both of which may contribute to the checkpoint mechanisms that eliminate persistently asynaptic meiocytes 8–10,13,15,17–23. Key outstanding questions include: How are HORMAD1 and HORMAD2 recruited to chromosome axes? What is the functional significance of their axial localization? And do soluble, off-axis pools of HORMADs contribute to ATR activation and DDR-like responses during asynaptic meiosis? Created in BioRender. Richter, C. (2026) https://BioRender.com/69j1otg.

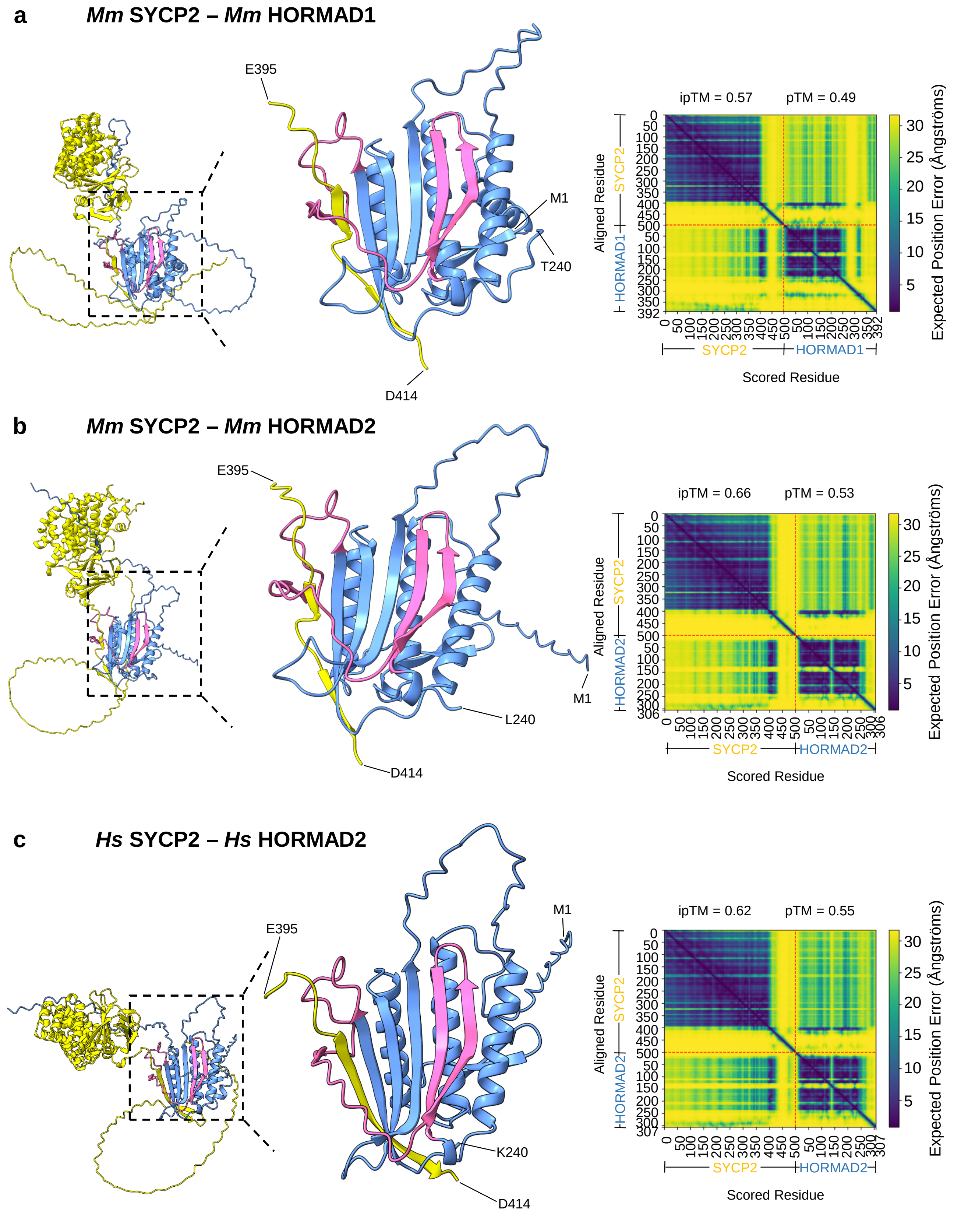

Supplementary Figure 2. AlphaFold 3 models for HORMAD1 and HORMAD2 interactions with a predicted closure motif in SYCP2.

a-c ChimeraX[24]-visualized AlphaFold 3 models[25] of complexes between a fragment of (**a-b**) mouse (*Mus musculus* (*Mm*)) SYCP2 (amino acids (aa) 1-500) and full-length HORMAD1 (aa 1-392) (**a**) or HORMAD2 (aa 1-306) (**b**), (**c**) human (*Homo sapiens* (Hs)) SYCP2 (amino acids (aa) 1-500) and full-length HORMAD2 (aa 1-307). Colors: SYCP2 is yellow, HORMA domain safety belts (HORMAD1, aa 180-230; HORMAD2, aa 184-235 in mouse; HORMAD2, aa 186-237 in human) are pink, and the remaining HORMAD regions are blue. Insets of the full models (far left) are enlarged (middle), showing the predicted closure motif of SYCP2 (aa 395-414) wrapped by the safety belts on the surface of the HORMA domains. Right panels show predicted aligned error (PAE) plots with ipTM and pTM scores.

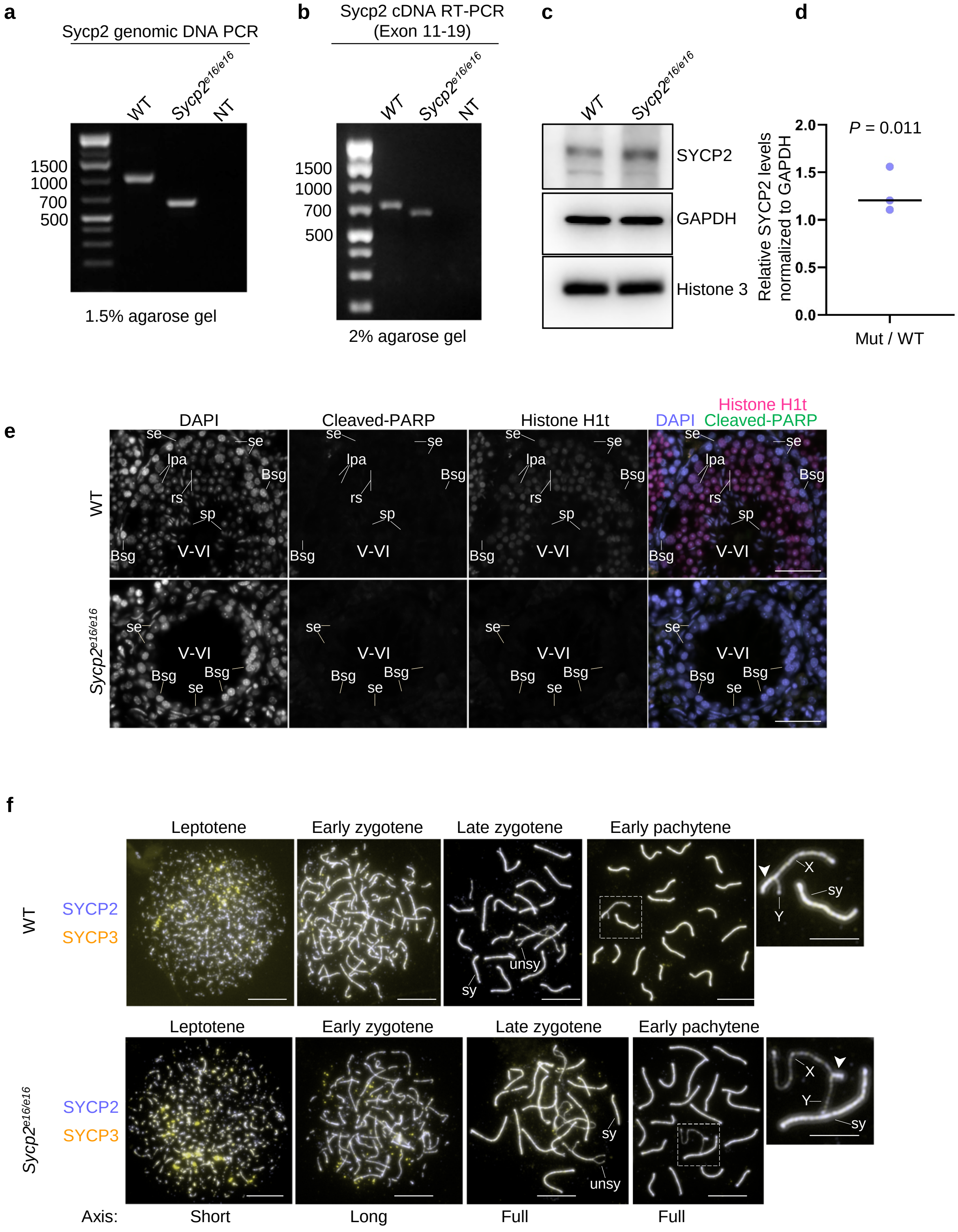

Supplementary Figure 3. Spermatogenic failure despite efficient axis formation in *Sycp2*$^{e16/e16}$.

a Agarose gel electrophoresis of PCRs using genomic DNA as a template and primers annealing to genomic loci flanking the 16th exon of *Sycp2*. Predicted PCR product sizes: 1057 bp in WT, and 643 bp in *Sycp2*$^{e16/e16}$. **b** Agarose gel electrophoresis of reverse transcription PCRs using total testis RNA as a template from 13 days post-partum (dpp) mice, amplifying sequences between the 11th and 19th exons of *Sycp2*. Predicted PCR product sizes: 713 bp in WT, and 638 bp in *Sycp2*$^{e16/e16}$. **a-b** No-template PCRs (NT) are shown. **c** SDS-PAGE immunoblots of total protein extracts from testes of WT and *Sycp2*$^{e16/e16}$ mice at 13 dpp. GAPDH (cytoplasmic marker) and histone H3 (chromatin marker) serve as loading controls. **d** Quantification of total SYCP2 protein abundance in testes of 13 dpp mice. SYCP2 immunoblot signals from testis extracts of *Sycp2*$^{e16/e16}$ mice were normalized to corresponding signals from wild-type controls. Bar indicates mean = 1.29 from n=3 experiments; two-tailed one-sample *t*-test, *P* = 0.0111. **e** DNA staining (DAPI) and immunostaining of cleaved PARP and histone H1t in testis cryosections from adult mice, showing seminiferous tubules in epithelial cycle stages V-VI (see Methods for staging) (n=4 biological replicates). The images show the presence of pachytene spermatocytes and post-meiotic cells in the WT testis section and their absence in the *Sycp2*$^{e16/e16}$ testis section, consistent with spermatocyte elimination at epithelial cycle stage IV in the mutant, as shown in Fig. 1d. Sertoli cells (Se), type B spermatogonia (Bsg), late pachytene spermatocytes (Lpa), round spermatids (rs), and sperm (sp) are marked. Scale bars, 50 µm. **f** Immunostained nuclear spreads of spermatocytes from adult mice. Morphology categories for axes (relevant to Fig. 1e) indicated at the bottom of the panel characterize the prophase stages indicated above each image (n=3 biological replicates). Unsynapsed (unsy) and synapsed (sy) regions of autosomes are marked in late zygotene. X and Y chromosomes and the pseudoautosomal region (PAR; white arrowheads) are marked in enlarged insets of early pachytene images. Scale bars, 10 µm (cell) and 5 µm (insets). Source data are provided as a Source Data file.

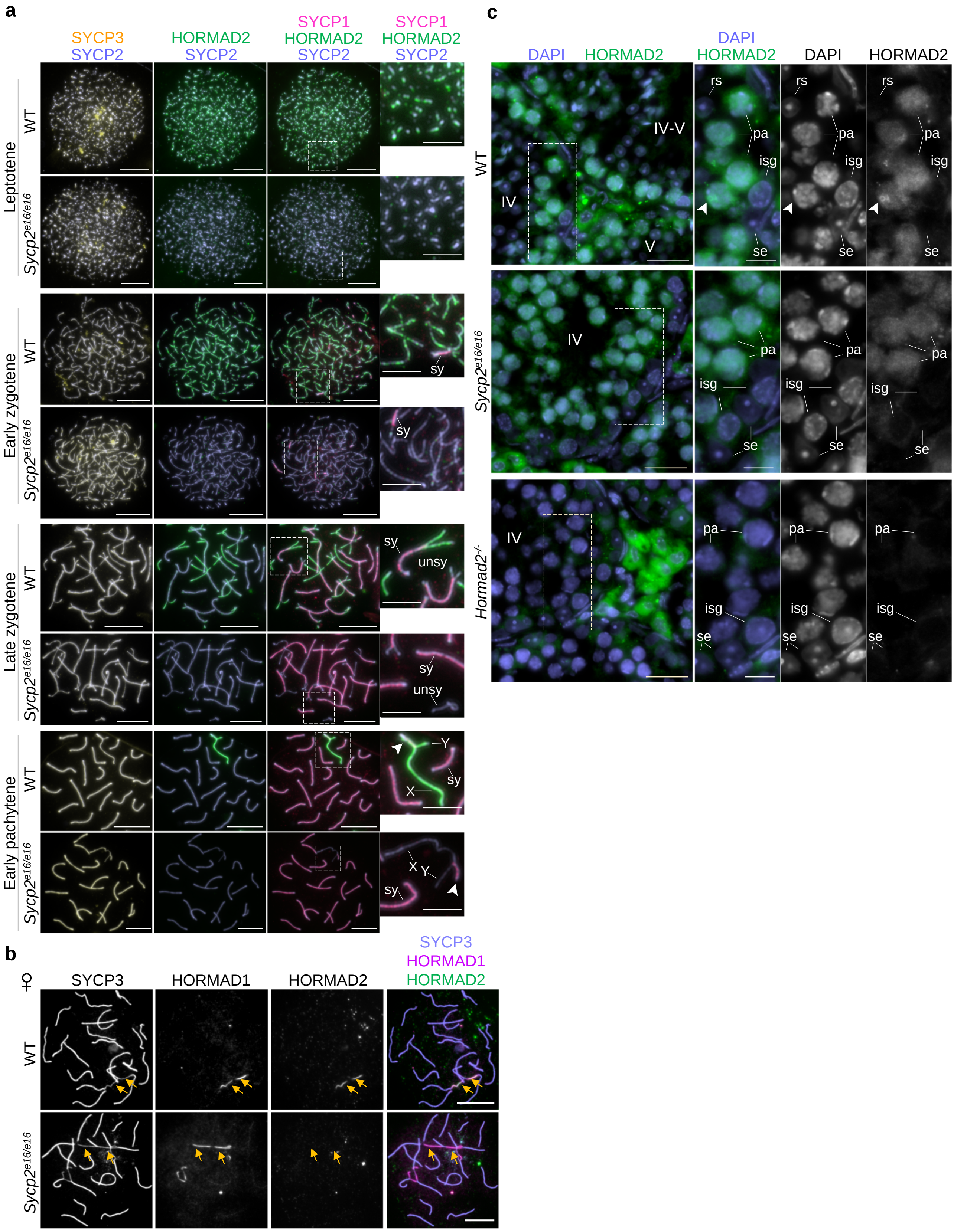

Supplementary Figure 4. HORMAD2 is present in *Sycp2$^{e16/e16}$* spermatocytes but depleted from the chromosome axes.

a-b Immunostaining in nuclear spreads of spermatocytes **(a)** from adult mice (n=5 biological replicates) or oocytes (n=4 biological replicates) **(b)** from 17 dpc foetuses. Scale bars, 10 µm (cell) and 5 µm (inset). Images with matched exposure and levelling are shown within each stage. The channels were differentially levelled between different stages to optimize viewing. **a** X and Y chromosomes, the pseudoautosomal region (PAR; white arrowheads), and examples of synapsed (sy) and unsynapsed (unsy) regions of autosomes are marked in enlarged insets. **b** Unsynapsed axes are marked by yellow arrows**. c** DNA staining (DAPI) and immunostaining of HORMAD2 in cryosections of testes from adult mice. Stages of the seminiferous epithelial cycle are indicated (see 'Methods' for staging). Sertoli cells (se), intermediate spermatogonia (isg), pachytene (pa) spermatocytes, and round spermatids (rs), are marked in the insets. White arrowheads mark the sex body, identified by enriched HORMAD2 signal in WT spermatocytes**.** Note the absence of this enrichment despite presence of nuclear HORMAD2 in pachytene spermatocytes in the *Sycp2$^{e16/e16}$* testis. Scale bars, 25 µm (section) and 10 µm (inset).

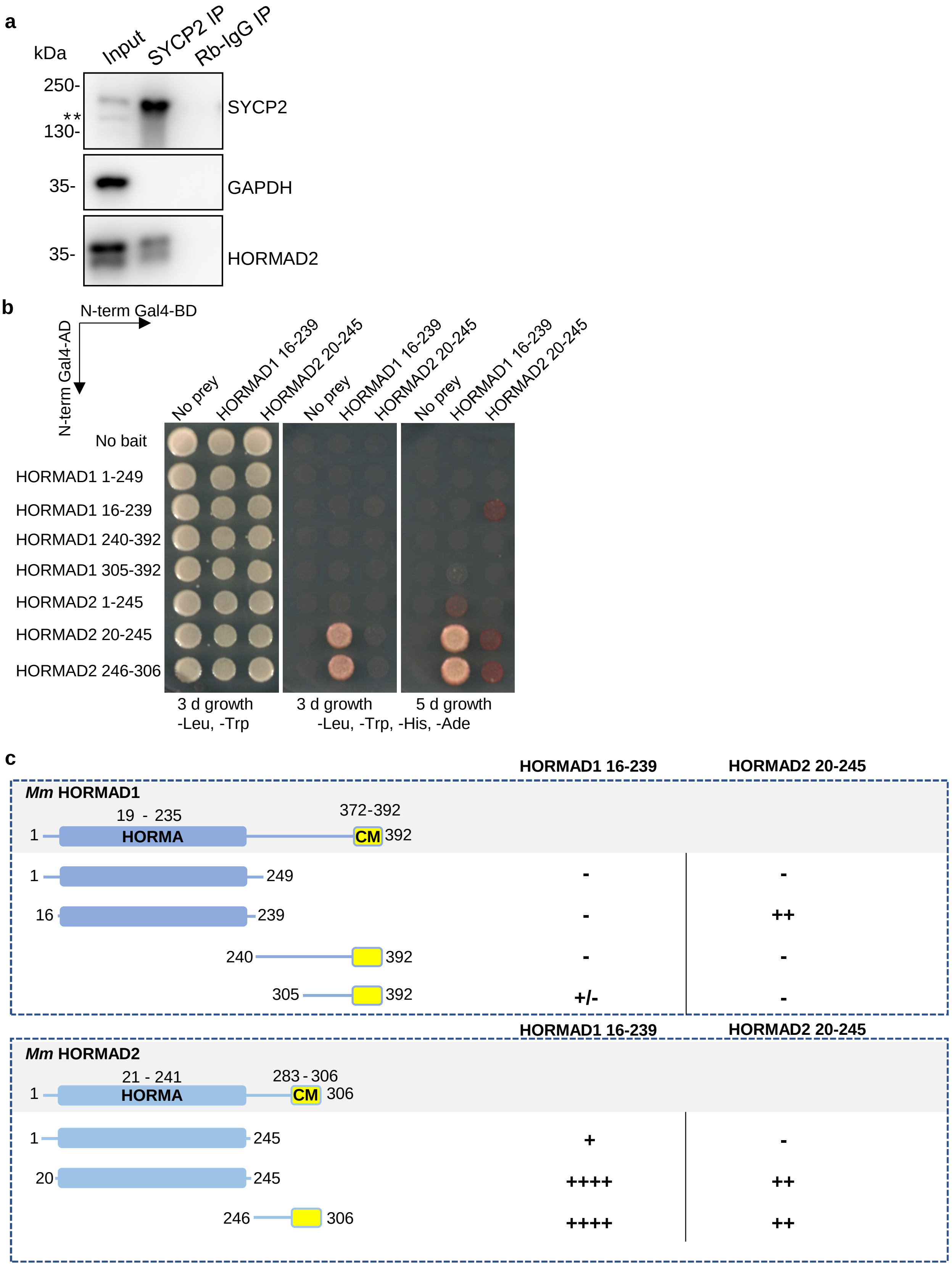

Supplementary Figure 5. Y2H interactions between HORMADs and complex formation between SYCP2 and HORMAD2.

a SDS-PAGE immunoblot analysis of protein extracts from testes of WT juvenile mice (n=2 biological replicates) (11 dpp). Total lysate (Input), and immunoprecipitates with anti-SYCP2 (SYCP2 IP) or non-specific rabbit IgG (Rb-IgG IP) antibodies are shown. Asterisks (**) mark an aspecific band that is observed in total protein extracts when analysed in 10% PAGE (see also Fig. 2d-e) but undetectable in the immunoprecipitated product. **b** Yeast two-hybrid interaction assays testing interactions between fragments corresponding to distinct domains of HORMAD1 and HORMAD2 (n=3 independent experiments). Amino acid (aa) positions of fragment ends are indicated. Yeast cultures are shown after 3 and 5 days of growth on dropout plates. For negative control, proteins of interest were tested in transformations where either the Gal4-binding domain (Gal4-BD) or the Gal4-activation domain (Gal4-AD) vectors were empty. **c** Schematics of HORMAD1 and HORMAD2 domain structures and summary of Y2H interactions between their HORMA domains and depicted protein fragments. Boxes mark the positions of HORMA domains and predicted closure motifs (CMs) as previously described [26]. Numbers represent amino acid positions. Strength of Y2H interactions is graded from no interaction (−) to very strong interaction (++++). Source data are provided as a Source Data file.

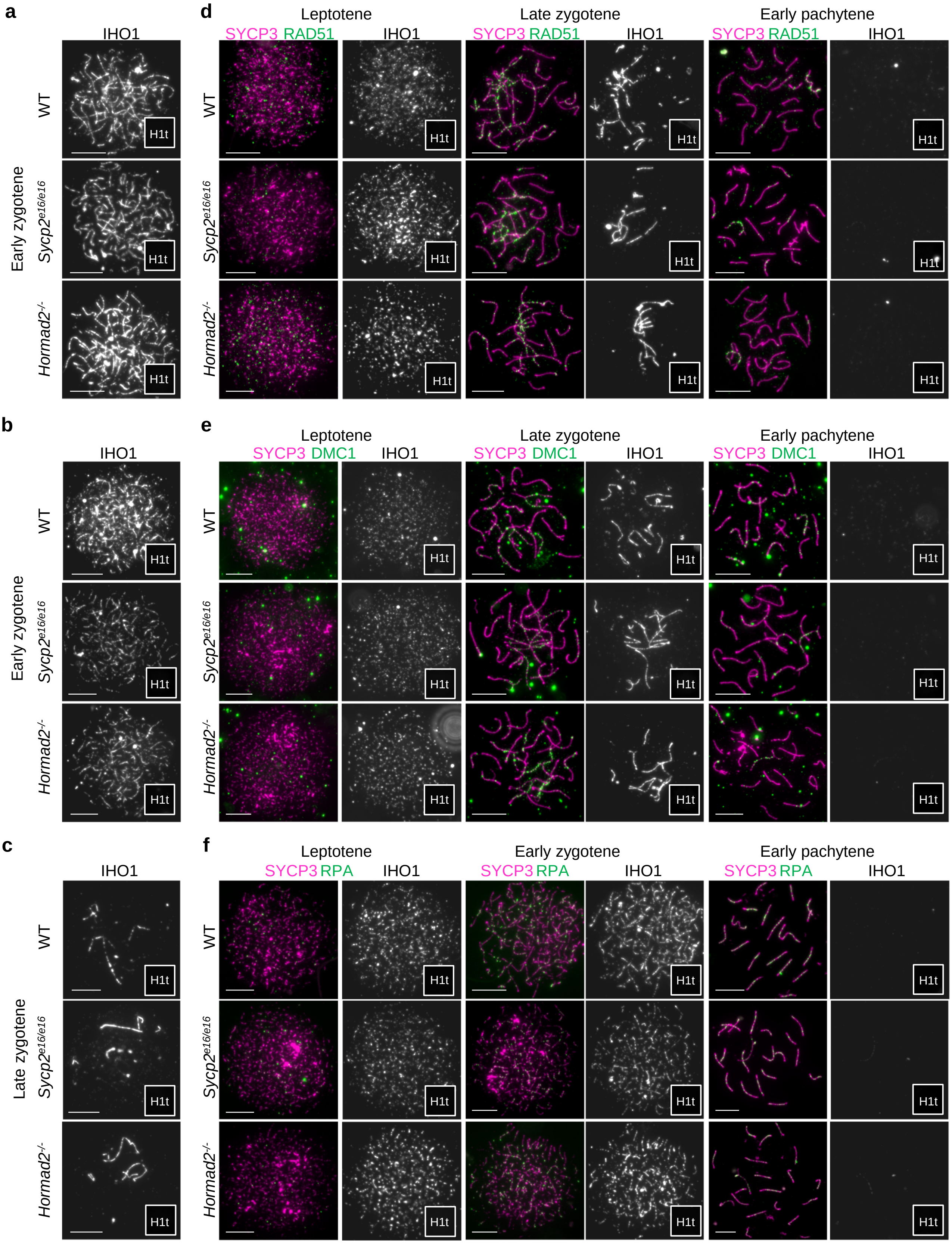

Supplementary Figure 6. Recombination foci in WT, *Sycp2*$^{e16/e16}$ and *Hormad2*$^{-/-}$ spermatocytes.

Immunostaining of nuclear surface spreads of spermatocytes from adult mice. For recombination markers (RAD51, n=4 biological replicates; DMC1 and RPA, n=3 biological replicates), images are shown with matched exposure and levelling across all genotypes and prophase stages (**d-f**). For stage markers (IHO1 and histone H1t), exposure and levelling are matched across genotypes for late zygotene and early pachytene; at other stages, exposure and levelling are matched across genotypes but differentially adjusted between stages for optimal viewing. SYCP3 was differentially levelled to optimize visualization. Histone H1t is shown in miniaturized images in the bottom right corners of full-size cell images. Panels **a-c** show IHO1 and histone H1t images in cells corresponding to Fig. 3d-f. Scale bars, 10 µm.

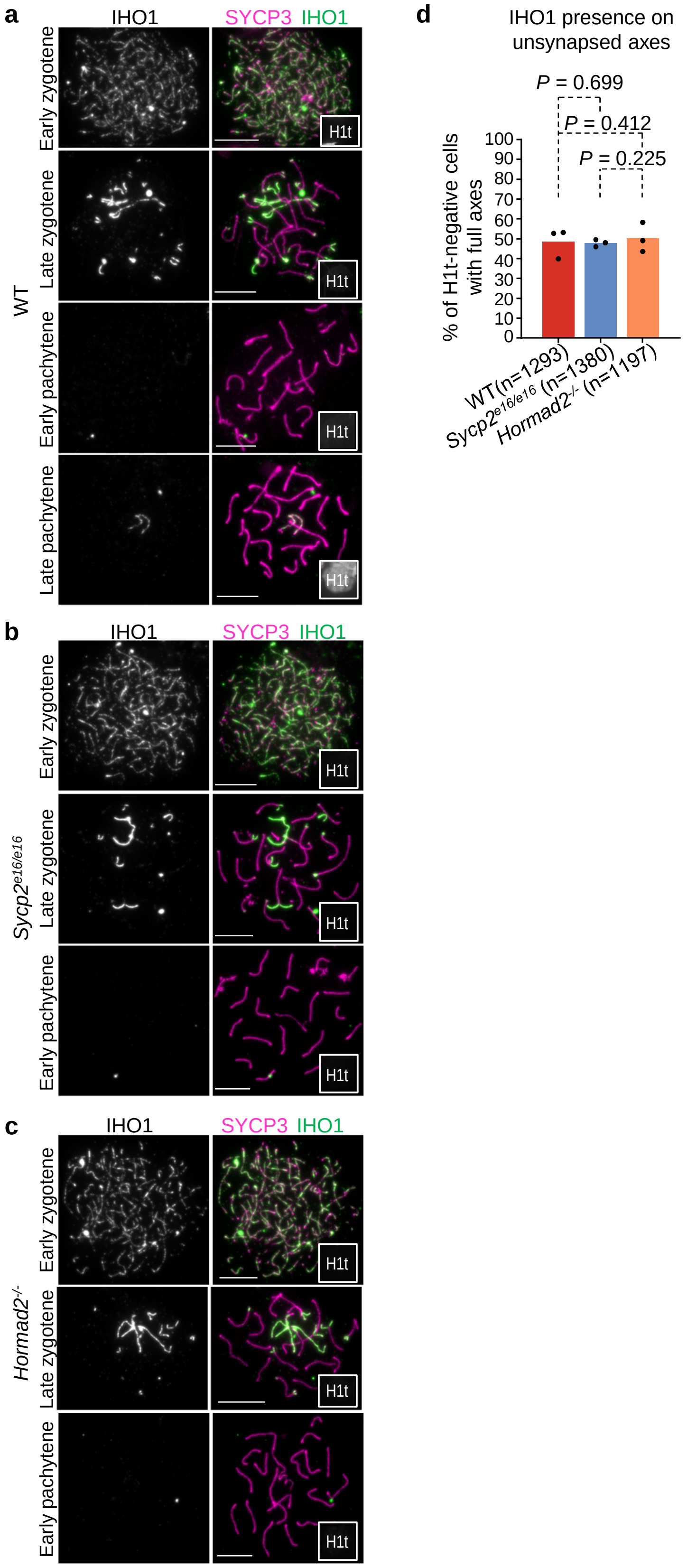

Supplementary Figure 7. IHO1 localization in WT, *Sycp2$^{e16/e16}$* and *Hormad2$^{-/-}$* spermatocytes.

a-c Immunostaining of nuclear surface spreads of spermatocytes from adult mice. Images with matched exposure and levelling are shown for stage markers IHO1 and histone H1t (miniaturized images in bottom right corner of full-size cell images) across genotypes and prophase stages (n=3 biological replicates). To illustrate IHO1 dynamics, early zygotene, late zygotene, and early pachytene are compared between WT **(a)**, *Sycp2*$^{e16/e16}$ **(b)**, and *Hormad2*$^{-/-}$ **(c)** genotypes; late pachytene is only shown in WT **(c)** because the mutants lack spermatocytes beyond mid-pachytene. SYCP3 was differentially levelled to optimize viewing. Scale bars, 10 µm. **d** Quantification of IHO1 localization on axes of spermatocytes that have fully developed axes and are negative for histone H1t (corresponding to late zygotene and early pachytene in wild-type spermatocytes). Graph shows data points and weighted averages (bars) of percentages of IHO1-positive spermatocytes (WT = 48.60%, *Sycp2*$^{e16/e16}$ = 47.90%, and *Hormad2*$^{-/-}$ = 50.33%) from three biological replicates. Number of analysed cells (*n*) per genotype is indicated. Likelihood-ratio test (chi-squared distribution) was used to determine whether the proportion of IHO1-positive cells among histone H1t-negative, fully-axis-displaying spermatocytes differed significantly between mutants and wild type. Source data are provided as a Source Data file.

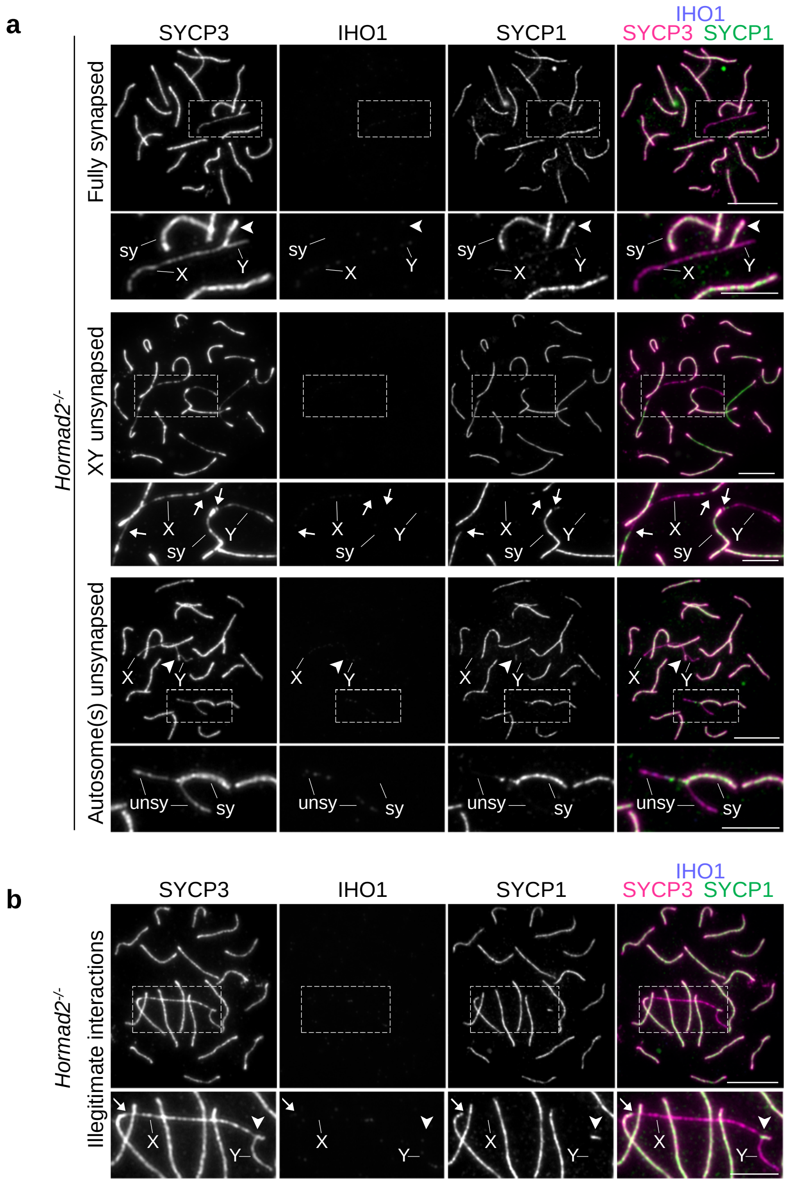

Supplementary Figure 8. *Hormad2*$^{-/-}$ spermatocytes display mild synapsis defects.

a-b Immunostained nuclear spreads of early pachytene *Hormad2*$^{–/–}$ spermatocytes from adult mice illustrating synapsis categories quantified in Fig. 4c-d (n=3 biological replicates). SYCP1 marks synapsed regions. Fully formed axes (SYCP3) and the absence of IHO1 identify the cells as early pachytene. Pseudoautosomal regions (PARs; white arrowheads in **a**-**b**), synapsed (sy) and unsynapsed (unsy) autosomal regions (**a**), and illegitimate interactions between the ends of the X or Y chromosomes and fully synapsed autosomes (white arrows in **a**-**b**) are indicated. Scale bars, 10 µm (whole cell) and 5 µm (insets).

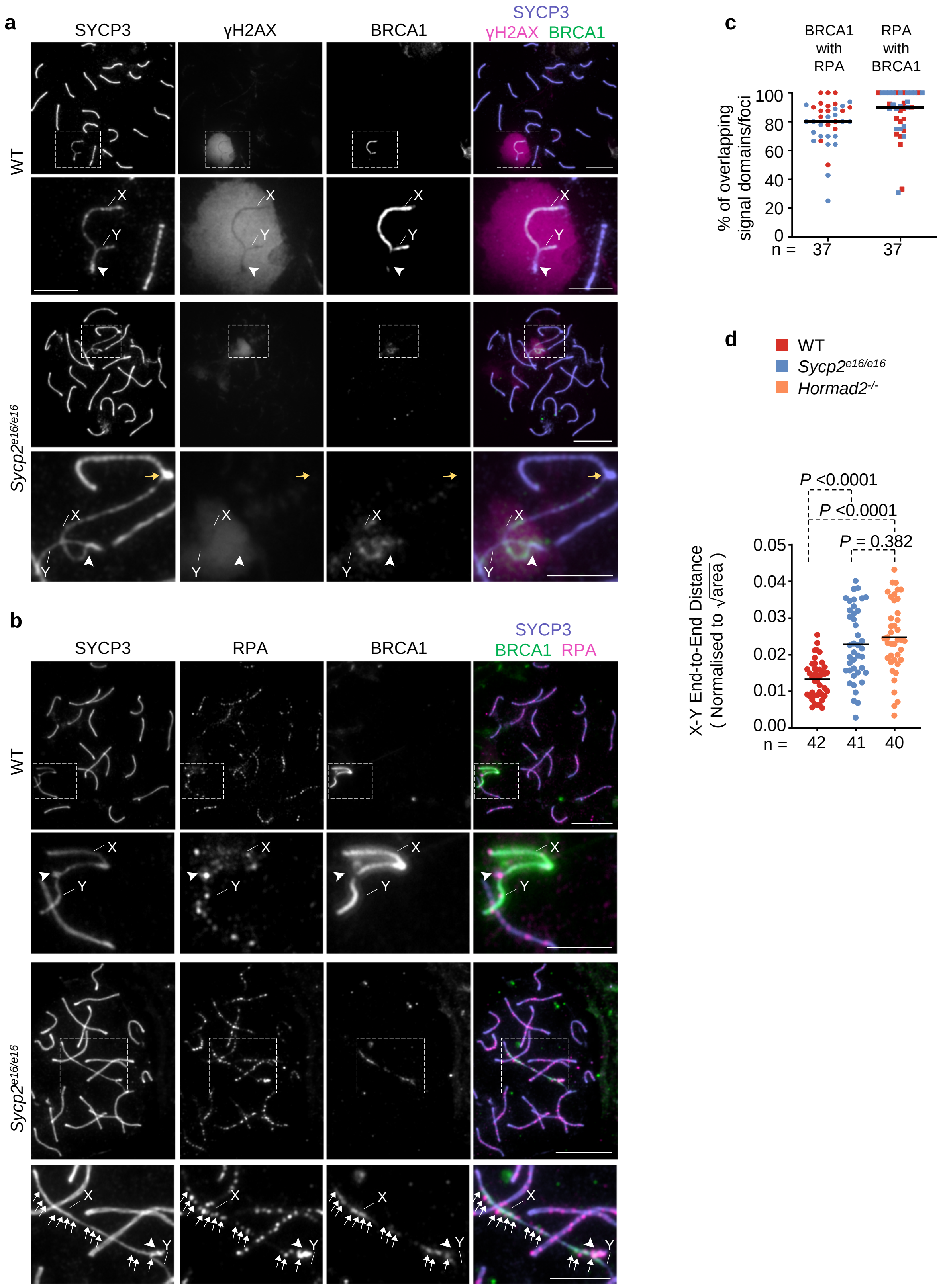

Supplementary Figure 9. The 16th exon of *Sycp2* is required for efficient BRCA1 accumulation on sex chromosomes.

a-b Immunostained nuclear surface spreads of early pachytene spermatocytes from adult mice (n=2 biological replicates). Images with matched exposure and levelling are shown for γH2AX, RPA and BRCA1 signals. X and Y chromosomes, PARs (white arrowheads in **a-b**), an illegitimate end-to-end association between the X chromosome and an autosome (yellow arrow in **a**), and sites of sex-chromosome-axis-associated RPA foci (arrows in **b**) are marked in enlarged insets. Scale bars, 10 µm (cells), 5 µm (insets). **c** Quantification of overlap between axis-associated RPA and BRCA1 signals on sex chromosomes in *Sycp2*$^{e16/e16}$ spermatocytes. The numbers of analysed cells (n) correspond to two experiments (differentiated by blue and red colors); medians (bars) are 80% (BRCA1 colocalizing with RPA), and 90% (RPA colocalizing with BRCA1). **d** Quantification of X–Y end-to-end distance in WT, *Sycp2*$^{e16/e16}$ and *Hormad2*$^{-/-}$ early pachytene spermatocytes. Number of analysed cells (n) and means (bars) are shown from two experimental replicates. P values were calculated using a two-tailed unpaired t-test with Welch's correction. Source data are provided as a Source Data file.

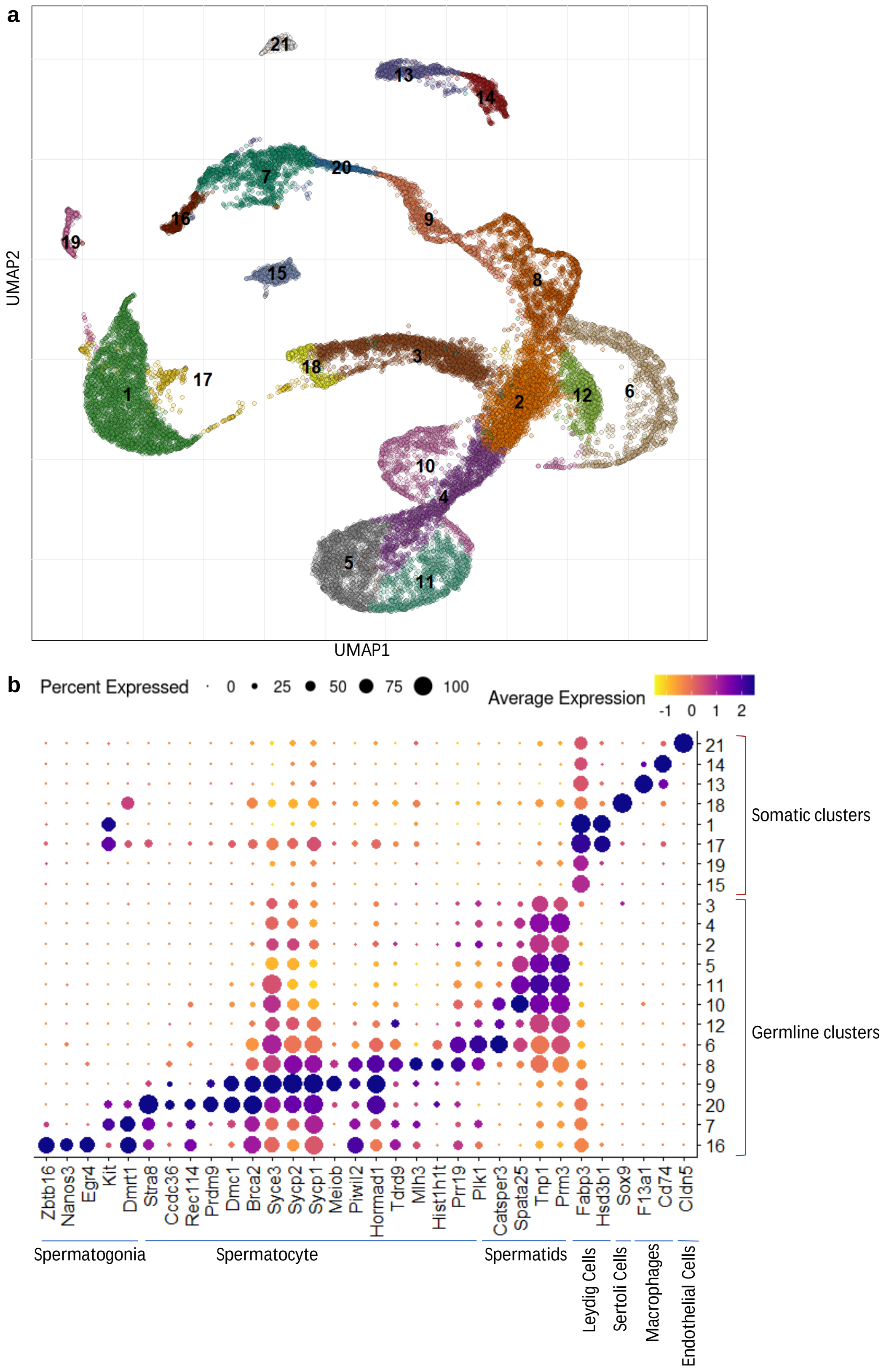

Supplementary Figure 10. Unsupervised clustering of pooled scRNAseq data from testes of WT, *Sycp2*$^{e16/e16}$, and *Hormad2*$^{-/-}$ mice.

a UMAP visualization of whole testis scRNA-seq data, showing cell populations identified by unsupervised Seurat-based clustering (cluster resolution parameter = 0.5). Pooled data are shown from testes of WT (four samples from two biological replicates), *Sycp2*$^{e16/e16}$ (two biological replicates), *Hormad2*$^{-/-}$ (two biological replicates) mice. The numbers represent clusters of distinct testicular cell populations as listed in **b**. **b** Dot plot heatmaps showing expression of selected marker genes. The x-axis lists representative marker genes and the cell types that primarily express them; the y-axis lists cluster numbers corresponding to **a**. Expression levels are represented as z-scores ranging from –1 to 2. The size and color intensity of each dot indicate the proportion of cells expressing the gene and the relative expression level, respectively. The full list of marker genes used for identifying spermatogenic and somatic clusters, and the distinguishing characteristics of the cell clusters are listed in Supplementary Data 1 and 2.

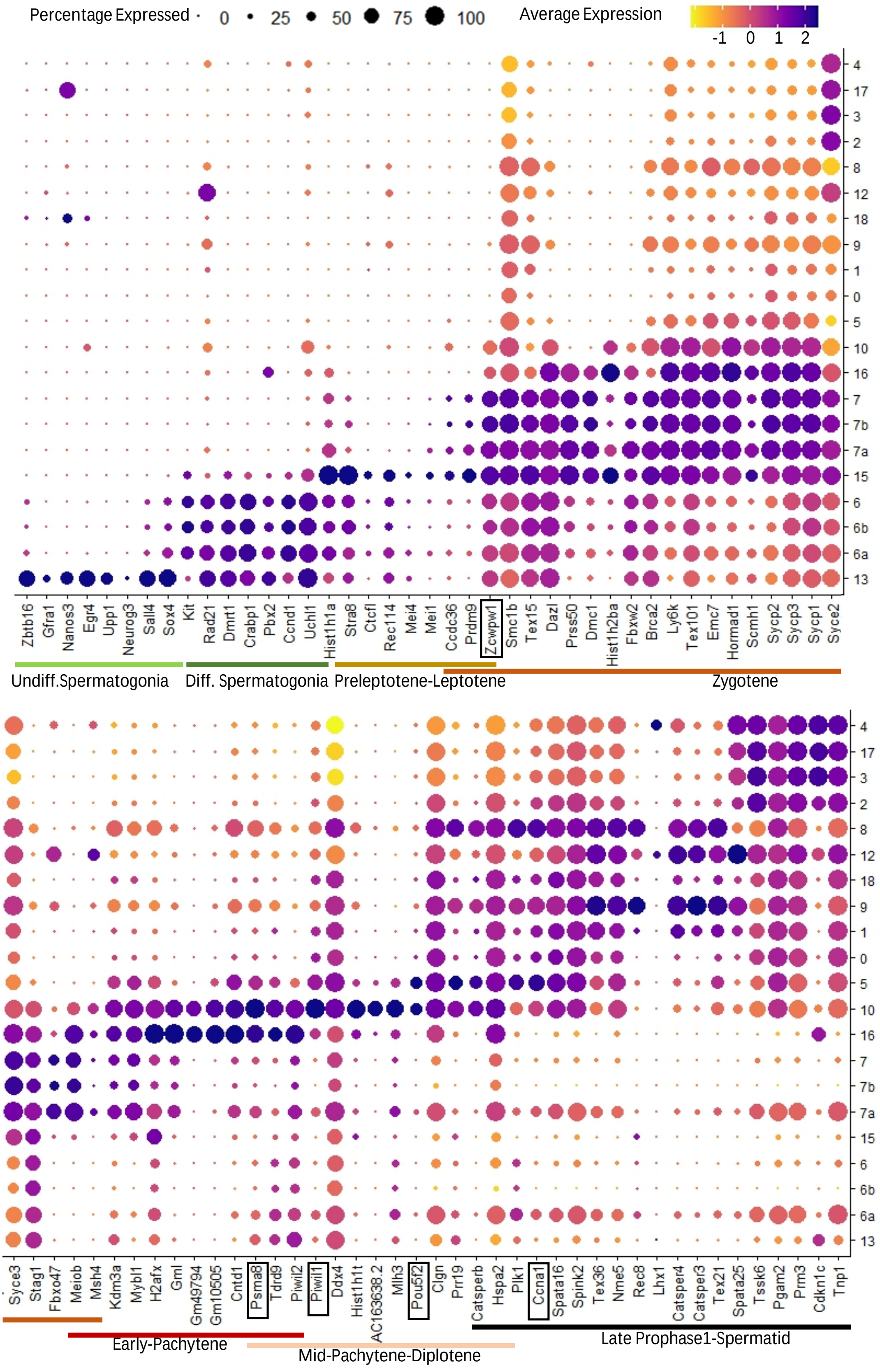

Supplementary Figure 11. Expression of marker genes in spermatogenic populations identified by supervised clustering.

Dot plot showing scRNA-seq–derived expression levels of all the marker genes used for supervised clustering and subsequent annotation of spermatogenic cell populations. Genes used exclusively for cell-type annotation are boxed. The x-axis lists marker genes; the y-axis lists cluster numbers corresponding to those in Fig. 6a–b. Clusters 6 and 7 are shown both as their constituent subclusters (6a/6b and 7a/7b) and as pooled clusters. In both cases, WT cells are preferentially enriched in subcluster “a” and mutant cells in subcluster “b,” despite highly similar expression profiles of stage-defining markers. Expression values are represented as z-scores ranging from –1 to 2. Dot size and color intensity indicate the proportion of cells expressing each gene and the relative expression level, respectively. Colored bars below the x-axis denote the cell populations predominantly expressing the corresponding markers. A complete list of marker genes used for clustering and their defining characteristics is provided in Supplementary Data 1 and 3.

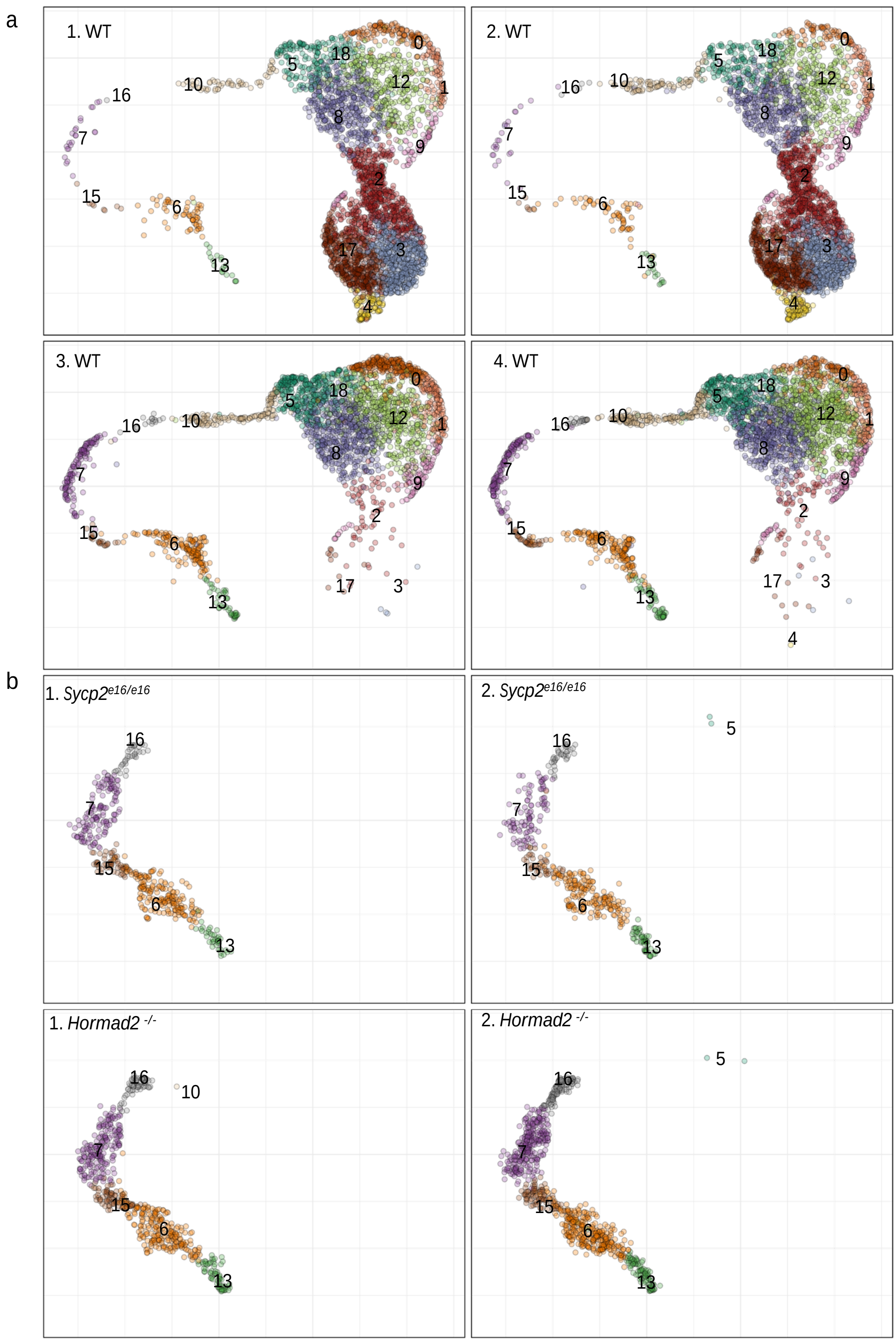

Supplementary Figure 12. scRNA-seq UMAPs of spermatogenic populations from individual WT, *Sycp2$^{e16/e16}$*, and *Hormad2$^{-/-}$* samples.

UMAP visualization of single-cell RNA sequencing data showing spermatogenic cell populations identified by supervised clustering using germline marker genes listed in Supplementary Fig. 10 (see also methods). Cluster numbers correspond to spermatogenic stages listed in Fig. 6b. **a** UMAPs for two biological replicates (left and right columns) of WT samples processed either without (top row) or with (bottom row) sperm and dead cell depletion. **b** UMAPs for two biological replicates (left and right columns) of *Sycp2$^{e16/e16}$* (top row), and *Hormad2$^{-/-}$* (bottom row) samples.

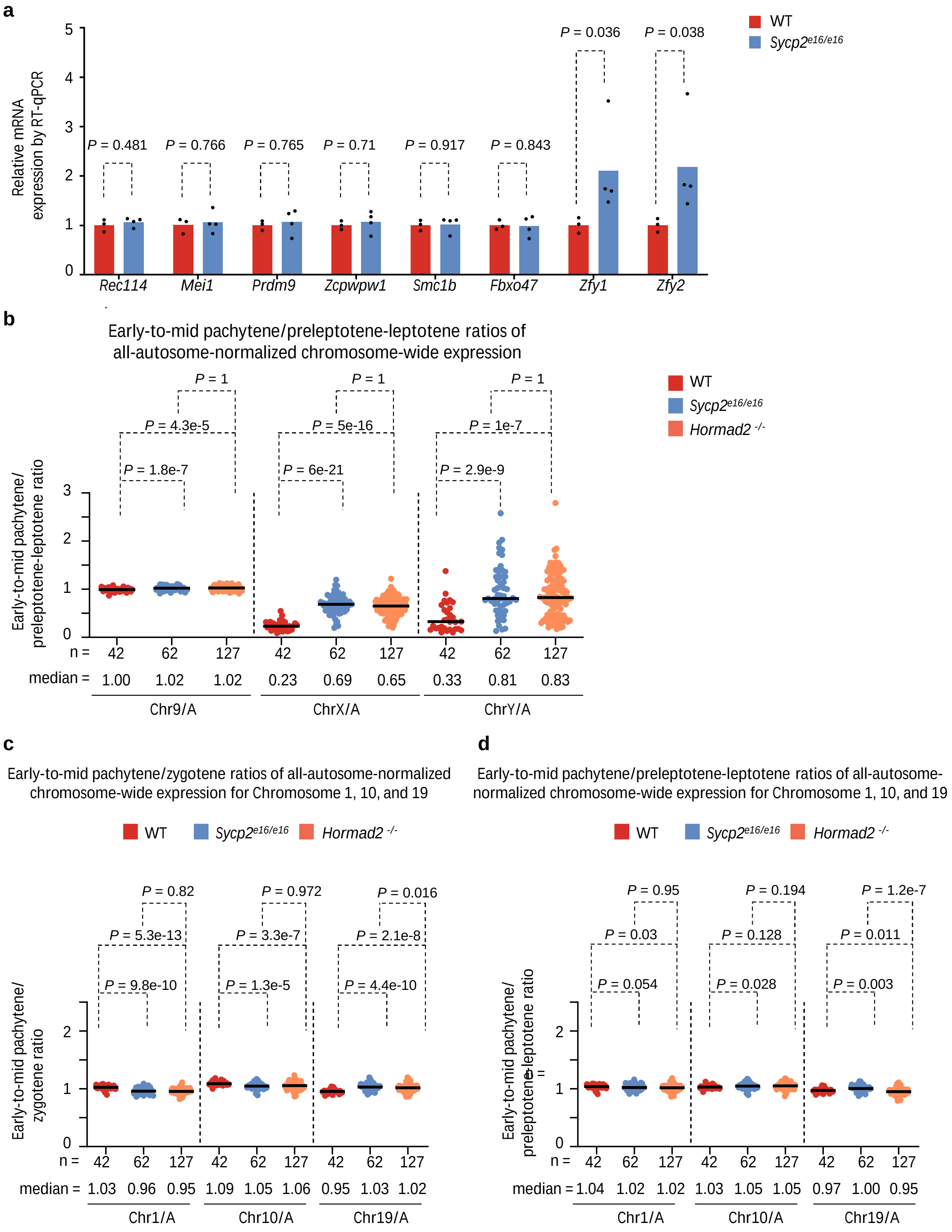

Supplementary Figure 13. RT-qPCR and scRNA-seq reveal impaired sex chromosome silencing in *Hormad2*$^{-/-}$ and *Sycp2*$^{e16/e16}$ spermatocytes.

a RT–qPCR quantification of transcript levels in *Sycp2*$^{e16/e16}$ testes, shown as fold change relative to WT following normalization to ribosomal housekeeping genes. Analysis includes transcripts of early prophase markers enriched in preleptotene-to-zygotene stages (*Rec114*, *Mei1*, *Prdm9*, *Zcpwpw1*, *Smc1b*, and *Fbxo47*), and the sex-chromosome-linked genes *Zfy1* and *Zfy2,* which are enriched in zygotene in WT. Total testicular RNA was isolated from 13-day-old juvenile mice. The same samples were used as in Fig. 6d-e. *Zfy1* and *Zfy2* RT-qPCRs were repeated alongside RT-qPCRs for the autosomal marker transcripts to permit direct comparisons. Individual data points and mean fold-change values (bars) from three (WT) or four (*Sycp2*$^{e16/e16}$) biological replicates are shown. *P* values, one-way ANOVA followed by Tukey's HSD test. **b** All-autosome-normalized chromosome-wide expression in early-to-mid pachytene cells relative to preleptotene-leptotene stages. Each data point represents normalized expression for chromosome 9 (Chr9/A), the X chromosome (ChrX/A), or the Y chromosome (ChrY/A) in early-mid pachytene cells divided by the median normalized expression of the same chromosome in preleptotene-leptotene cells (see Methods for details). *P* values were determined using the Wilcoxon rank-sum test. **c-d** All-autosome-normalized chromosome-wide expression in early-to-mid pachytene cells relative to zygotene (**c**) and preleptotene-leptotene (**d**) stages. Each data point represents all-autosome-normalized expression for chromosome 1 (Chr1/A), chromosome 10 (Chr10/A), or chromosome 19 (Chr19/A) in individual early-mid pachytene cells divided by the median normalized expression of the same chromosome in zygotene (**c**) or preleptotene-leptotene (**d**) cells. Data points represent individual cells; medians (black bars), and *P* values from the Wilcoxon rank-sum test are indicated. Source data are provided as a Source Data file.

Supplementary Table 1. Fertility in female *Sycp2* $^{e16/e16}$ and *Hormad2* $^{-/-}$ mice.

Quantification of pup numbers from crosses of wild-type (WT) male mice with WT, *Sycp2* $^{e16/e16}$ or *Hormad2* $^{-/-}$ female mice. Statistical significances were calculated by two-tailed unpaired t-test with Welch correction.

female age	female < 35 weeks			female >35 weeks		
female genotype	WT	*Sycp2* $^{e16/e16}$	Hormad2$^{-/-}$	WT	*Sycp2* $^{e16/e16}$	Hormad2$^{-/-}$
male genotype	WT					
breeding pairs	4	4	4	4	4	4
total breeding weeks	91	93	96	61	55	51
pups/breeding week	1.76	1.63	1.53	0.8	0.86	0.94
Comparison with WT (P values from unpaired t-test with Welch's correction)	x	0.6979	0.2595	x	0.6699	0.5453

Supplementary Table 2. **Fertility in female *Sycp2* $^{e16/e16}$ and *Hormad2* $^{-/-}$ mice**. Quantification of pup numbers from crosses of wild type (WT) male mice with WT, *Sycp2* $^{e16/e16}$ or *Hormad2* $^{-/-}$ female mice. To account for differences in breeding duration, total pup number and mean litter size were calculated over the same breeding period common to all breeding pairs (34.86 weeks per pair). Statistical significance was determined by two-tailed unpaired t-test with Welch's correction.

female genotype	WT	*Sycp2* $^{e16/e16}$	Hormad2$^{-/-}$
male genotype		WT	
breeding pairs	4	4	4
total pups/female	49.75	44.25	47.25
comparison with WT (P values from unpaired t-test with Welch's correction)	x	0.372	0.568
average litter size/female	7.1	7.66	7.03
comparison with WT (P values from unpaired t-test with Welch's correction)	x	0.144	0.799
average litter number/female	7	5.77	6.72
comparison with WT (P values from unpaired t-test with Welch's correction)	x	0.095	0.705

Supplementary Table 3. **Quantification of spermatocyte apoptosis in testes of *Sycp2* $^{e16/e16}$ mice.**
The percentage of seminiferous tubules containing cleaved PARP-positive cells at stage IV of the seminiferous epithelial cycle in WT and *Sycp2* $^{e16/e16}$ testes. The number of cleaved PARP-positive cells per stage IV seminiferous tubule is also indicated. P value for the proportion of PARP-positive tubules was calculated using a likelihood ratio test; analyses were based on two *Sycp2* $^{e16/e16}$ and three WT biological replicates.

Genotype	total number of scored stage IV tubules		Number of PARP positive cells/tubule: 0	1 to 3	4 to 6	>6	total >0	P value
WT	38	Percentage (numbers) of tubules	97.4 (37)	2.6 (1)	0	0	2.6 (1)	2.41E-15
Sycp2 $^{e16/e16}$	56		21.4 (12)	21.4 (12)	25 (14)	32.2 (18)	78.6 (44)	

Supplementary Table 4. Summary of single-cell RNA-seq quality metrics in wild-type and mutant testes samples. Key sequencing and mapping statistics for four wild-type (WT), two *Hormad2* $^{-/-}$, and two *Sycp2* $^{e16/e16}$ samples. Two biological replicates of WT samples were processed either without (1 and 2) or with (3 and 4) sperm and dead cell depletion.

		Samples							
		**1. WT**	**2. WT**	**3. WT**	**4. WT**	**1. *Hormad2*** $^{-/-}$	**2. *Hormad2*** $^{-/-}$	**1. *Sycp2*** $^{e16/e16}$	**2. *Sycp2*** $^{e16/e16}$
Metric	ID	CMO309_WT (no dead cell removal)	CMO312_WT (no dead cell removal)	CMO310_WT (with dead cell removal)	CMO311_WT (with dead cell removal)	CM0309 (no dead cell removal)	CM0310 (no dead cell removal)	CM0311 (no dead cell removal)	CM0312 (no dead cell removal)
	Number of sequencing rounds	1	1	1	1	2	2	2	2
	Cells numbers before quality control	3860	4089	3678	4118	3163	3048	2784	2796
	Confidently mapped antisense	1.24%	1.26%	1.43%	1.50%	1.32%	1.46%	1.28%	1.35%
	Confidently mapped to exonic regions	79.87%	79.59%	72.50%	70.05%	75.91%	71.88%	77.82%	75.60%
	Confidently mapped to genome	93.27%	93.28%	92.20%	92.05%	92.21%	91.64%	92.46%	92.35%
	Confidently mapped to intergenic regions	5.92%	5.82%	7.16%	7.58%	3.27%	3.80%	3.03%	3.30%
	Confidently mapped to intronic regions	7.48%	7.87%	12.55%	14.41%	13.03%	15.96%	11.62%	13.45%
	Confidently mapped to transcriptome	76.25%	76.01%	68.73%	66.38%	65.31%	64.17%	65.82%	64.98%
	Mapped to genome	95.87%	95.86%	95.46%	95.31%	94.83%	94.46%	95.03%	94.94%
	Median UMI counts per cell	14631	12706	8555	6984	6956	5759	7879	7337
	Median genes per cell	3332	3230	3408	3073	2640	2441	2798	2700
	Median reads per cell	39845	36440	32869	28510	17692	15109	19995	19117
	Number of reads from cells called	273,559,970	261,150,602	292,705,166	260,357,842	69,938,676	59,008,196	69,298,650	65,718,657
	Total number of genes detected	26056	26009	26726	26745	21811	21998	21515	21973
	Cell numbers in input for unsupervised clustering after quality control	3703	3912	3402	3837	2355	2144	2162	2015
	Cell numbers in spermatogenic populations subjected to supervised clustering	3439	3501	2634	2872	455	417	643	881

Supplementary Table 5. Primers used for RT-qPCR in Figure 6d-e and Supplementary Figure 13a.

Primer Name	sequence (5'-3')
Zfy1_F	CAGATTGTGTTTCTGAAGCAGTCT
Zfy1_R	TCCTGACTCTGCATTCATGG
Zfy2_F	TCTGGAGCAGCAAGATGATG
Zfy2_R	TGCACACCTTGATAACTTCTGG
Rsp16_F	AATGGGCTCATCAAGGTGAACGGA
Rsp16_R	TTCACACGGACCCGAATATCCACA
S12_F	CTCGCATCCAACTGTGATGAG
S12_R	CTTGCACTTGAAGTATTCCTCG
s9_F	GGCCAAATCTATTCACCATGC
s9_R	TAATCCTCTTCCTCATCATCAC
Rec114_F	TCGTGTCCAGTTTAGTGGGG
Rec114_R	TCGATGTGTCACTGAGGTCC
Mei1_F	CATCTTTCGTCCTCCAGCGA
Mei1_R	CACTGCCTCGATCCCATACA
Prdm9_F	TCTGCACTTTGGCCCCTATG
Prdm9_R	GGGCACAGTTCACATACCTCAT
Zcpwpw1_F	ACCTTTGCTCCACCTACACAA
Zcpwpw1_R	CACAGGAAGAAGTCTCCAAGCA
Smc1b_F	CGTCTACCCAGAGCACAATGA
Smc1b_R	CGAGACTGGGGAGACTTTGG
Fbxo47_F	AATGCACGCCTCCTAATCCT
Fbxo47_R	CTCTTTCTCACACACCAAAGCC

Supplementary Methods

Reagents and resources

REAGENT or RESOURCE	SOURCE	IDENTIFIER
Antibodies		
Chicken polyclonal anti-SYCP3	[27]	N/A
Mouse monoclonal anti-SYCP3	[28]	N/A
Chicken polyclonal anti-IHO1	[29]	N/A
Rabbit polyclonal anti-IHO1	[29]	N/A
Guinea polyclonal pig anti-HORMAD1	[3]	N/A
Rabbit polyclonal anti-HORMAD1	[3]	N/A
Guinea pig polyclonal anti-HORMAD2	[1]	N/A
Guinea pig polyclonal anti-Histone H1t	[30]	N/A
Guinea pig polyclonal anti-Histone H1t	This study	N/A
Chicken polyclonal anti-SYCP1	[30]	N/A
Rabbit polyclonal anti-BRCA1	This study	N/A
Rabbit polyclonal anti-NOBOX	This study	N/A
Rabbit polyclonal anti-SYCP1	Abcam	Cat# ab15090 RRID: AB_301636
Rabbit polyclonal anti-SYCP2	This study	N/A
Guinea pig polyclonal anti-SYCP2	This study	N/A
Rabbit polyclonal anti-cleaved PARP (Asp214)	Cell signaling	Cat# 9544; RRID: AB_2160724
Mouse monoclonal anti-GAPDH	Santa Cruz	Cat# sc-32233 RRID: AB_627679

Rabbit polyclonal anti-Histone H3	Abcam	Cat# ab1791<br>RRID: AB_302613
Mouse monoclonal anti-DMC1 (2H12/4)	Abcam	Cat# ab11054;<br>RRID: AB_297706
Guinea pig polyclonal anti-DMC1	This study	N/A
Rabbit polyclonal anti-Rad51	Abcam	Cat# ab176458;<br>RRID: AB_2665405
Rabbit monoclonal anti-RPA32/RPA2 (EPR2877Y)	Abcam	Cat# ab76420;<br>RRID: AB_1524336
Mouse monoclonal anti-phospho-Histone H2A.X (Ser139)	Millipore	Cat# 05-636;<br>RRID: AB_309864
Goat polyclonal anti-ATR (N-19)	Santa Cruz	Cat# sc-1887;<br>RRID: AB_630893
Mouse monoclonal anti-Pol II	Santa Cruz	Cat# sc-56767<br>RRID: AB_785522
Mouse monoclonal anti-SYCP3	Abcam	Cat# ab 97672<br>RRID: AB_10678841
Goat anti-rabbit IgG-HRP	Jackson ImmunoResearch	Cat# 111-035-003;<br>RRID: AB_2313567
Goat anti-guinea pig IgG-HRP	Jackson ImmunoResearch	Cat# 706-035-148;<br>RRID: AB_2340447
Goat anti-mouse IgG-HRP	Jackson ImmunoResearch	Cat# 115-035-003;<br>RRID: AB_10015289
Goat anti-Rabbit IgG-AF405	Thermo Fisher Scientific	Cat# A-31556;<br>RRID: AB_221605

Goat anti-rabbit IgG-AF488	Thermo Fisher Scientific	Cat# A-11034; RRID: AB_2576217
Goat anti-rabbit IgG- AF568	Thermo Fisher Scientific	Cat# A-11036; RRID: AB_10563566
Goat anti-Rabbit IgG- AF647	Thermo Fisher Scientific	Cat# A-21244; RRID: AB_2535812
Donkey anti-guinea pig IgG-DyLight™405	Jackson ImmunoResearch	Cat# 706-475-148; RRID: AB_2340470
Goat anti-guinea pig IgG-AF488	Thermo Fisher Scientific	Cat# A-11073; RRID: AB_2534117
Goat anti-guinea pig IgG-AF568	Thermo Fisher Scientific	Cat# A-11075; RRID: AB_2534119
Goat anti-guinea pig IgG-AF647	Thermo Fisher Scientific	Cat# A-21450 RRID: AB_2735091
Donkey anti-guinea pig IgG-AF647	Jackson ImmunoResearch	Cat# 706-605-148 RRID: AB_2340476
Goat anti-mouse IgG-AF405	Thermo Fisher Scientific	Cat# A-31553; RRID: AB_221604
Goat anti-mouse IgG-AF488	Thermo Fisher Scientific	Cat# A-11029; RRID: AB_2534088
Goat anti-mouse IgG-AF568	Thermo Fisher Scientific	Cat# A-11031; RRID: AB_144696

Goat anti-chicken IgY-AF405	Abcam	Cat# ab175675 RRID: AB_2810980
Goat anti-chicken IgY-AF488	Thermo Fisher Scientific	Cat# A-11039; RRID: AB_2534096
Goat anti-chicken IgY-AF568	Thermo Fisher Scientific	Cat# A-11041; RRID: AB_2534098
Goat anti-rat IgG-AF488	Thermo Fisher Scientific	Cat# A-11006; RRID: AB_2534074
Goat anti-rabbit IgG-AF647	Thermo Fisher Scientific	Cat# A-21245; RRID: AB_2535813
Goat anti-chicken IgY-AF647	Thermo Fisher Scientific	Cat# A-21449; RRID: AB_2535866
Donkey anti-rabbit IgG-AF647	Jackson ImmunoResearch	Cat# 711-495-152; RRID: AB_2315775
Bovine anti-goat IgG-RRX	Jackson ImmunoResearch	Cat# 805-295-180; RRID: AB_2340881
Donkey anti-mouse IgG-AF350	Thermo Fisher Scientific	Cat# A-10035; RRID: AB_2534011
Donkey anti-guinea pig IgG-AF488	Jackson ImmunoResearch	Cat# 706-545-148; RRID: AB_2340472
Donkey anti-rabbit IgG-DyLight™ 488	Jackson ImmunoResearch	Cat# 711-485-152; RRID: AB_2492289

Chemicals, peptides, and recombinant proteins		
		Cat#<br>Cas#
6xHis-tagged SYCP2 C-terminus	This study	N/A
6xHis-tagged H1t C-terminus	This study	N/A
6xHis-tagged BRCA1 C-terminus	This study	N/A
6xHis-tagged NOBOX	This study	N/A
6xHis-tagged DMC1	This study	N/A
Critical commercial assays		
NHS-activated Sepharose 4 Fast Flow beads	Cytiva/GE Healthcare	Cat# 17-0906-01
Kits		
NucleoSpin RNA kit	Macherey-Nagel	Cat# 740955.50
First Strand cDNA Synthesis Kit	Thermo Fisher Scientific	Cat# K1612
iTaq Universal SYBR Green Supermix	Bio-Rad	Cat# 1725120
Experimental models: Organisms/strains		
Y2HGold Yeast strain	Clontech	Cat# 630498
E. coli strain BL21(DE3) pLysS		
E. coli strain BL21 *tRNA*		
E. coli strain Rosetta2(DE3)pLysS		
Mouse/ *Sycp2*$^{+/+}$ and *Sycp2*$^{e16/e16}$	This study	
Mouse/ *Hormad2*$^{-/-}$	[2]	
Mouse/ *Iho1*$^{+/+}$ and *Iho1*$^{-/-}$	[29]	
Mouse/ *Hormad1*$^{+/+}$ and *Hormad1*$^{-/-}$	[3]	
Mouse/ *Spo11*$^{+/+}$ and *Spo11*$^{-/-}$	[31]	
Mouse/ *Dmc1*$^{+/+}$ and *Dmc1*$^{-/-}$	[32]	
Mouse/ *Sycp1*$^{+/+}$ and *Sycp1*$^{-/-}$	[33]	
Mouse/ *Spo11*$^{-/-}$ *Sycp2*$^{+/+}$ and *Spo11*$^{-/-}$ *Sycp2*$^{e16/e16}$		

Mouse/ *Dmc1*$^{-/-}$ *Sycp2*$^{e16/e16}$	This study	
Mouse/ *Iho1*$^{-/-}$ *Sycp2*$^{e16/e16}$	This study	
Mouse/ *Sycp1*$^{+/+}$ *Sycp2*$^{+/+}$, *Sycp1*$^{-/-}$ *Sycp2*$^{+/+}$ and *Sycp1*$^{-/-}$ *Sycp2*$^{e16/e16}$	This study	
Oligonucleotides		
Sycp2_Fwd: TGCAAGGCTTCTCTGTTCCT	Eurofins Genomics	N/A
Sycp2_Rvs: TGCCTTTACAGTGGCTGCTT	Eurofins Genomics	N/A
crRNA1: TTATGTCAGACCAGGCTTAG	Integrated DNA Technologies	N/A
crRNA2: CATGTACTTTTTGATGCAAG	Integrated DNA Technologies	N/A
crRNA3: TTTTGATGCAAGTGGATCAC	Integrated DNA Technologies	N/A
crRNA4: AACTGAAATACCTTGTTTTC	Integrated DNA Technologies	N/A
crRNA5: GCAGAATTTGCTTACAAACA	Integrated DNA Technologies	N/A
crRNA6: AGGTGTAAACAGTTGACCTA	Integrated DNA Technologies	N/A
Trans-activating CRISPR RNA	Integrated DNA Technologies	N/A
To amplify C-terminus of SYCP2, SYCP2C_LICfwd: CACCACCACCACAGGGTATCAGG TCCCAGTCAACGTGG	Eurofins Genomics	N/A
To amplify C-terminus of SYCP2, SYCP2C_LICrvs: TGAGGAGAAGGCGCGTCATGCAT CATTTCCTTCATGAGCC	Eurofins Genomics	N/A

To amplify C-terminus of H1t, H1tcterm_fwd: ATCACCACCACCACA GG CTCAGTAAGAAGGCGGCTTCTG	Eurofins Genomics	N/A
To amplify C-terminus of H1t, H1tcterm_rvs: TGAGGAGAAGGCGCGTCACTTCC TCCCTGCTGCC	Eurofins Genomics	N/A
Software and algorithms		
ImageJ	[34]	
Fiji Suite for ImageJ	[35]	
Adobe Photoshop CC 19	Adobe	
Cell Ranger V6.1	[36]	
R	[37]	
Seurat v5	[38]	
scCustomize	[39]	
Ggplo2	[40]	
lmerTest Package	[41]	
Lme4 package	[42]	
CHOPCHOP version 2	[43,44]	
GT-Scan	[45]	
CFX Maestro software		Cat# 12013758
Alphafold 3	[25]	
ChimeraX [24] version 1.7.1		
Other		
salmon sperm	Thermo Fisher Scientific	Cat# AM9680

Supplementary References

1. Wojtasz, L. *et al.* Mouse HORMAD1 and HORMAD2, Two Conserved Meiotic Chromosomal Proteins, Are Depleted from Synapsed Chromosome Axes with the Help of TRIP13 AAA-ATPase. *PLoS Genetics* 5, e1000702; 10.1371/journal.pgen.1000702 (2009).
2. Wojtasz, L. *et al.* Meiotic DNA double-strand breaks and chromosome asynapsis in mice are monitored by distinct HORMAD2-independent and -dependent mechanisms. *Genes & development* 26, 958–973; 10.1101/gad.187559.112 (2012).
3. Daniel, K. *et al.* Meiotic homologue alignment and its quality surveillance are controlled by mouse HORMAD1. *Nature Cell Biology* 13, 599–610; 10.1038/ncb2213 (2011).
4. Ravindranathan, R., Raveendran, K., Papanikos, F., San-Segundo, P. A. & Tóth, A. Chromosomal synapsis defects can trigger oocyte apoptosis without elevating numbers of persistent DNA breaks above wild-type levels. *Nucleic acids research* 50, 5617–5634; 10.1093/nar/gkac355 (2022).
5. Kogo, H. *et al.* HORMAD2 is essential for synapsis surveillance during meiotic prophase via the recruitment of ATR activity. *Genes to Cells* 17, 897–912; 10.1111/gtc.12005 (2012).
6. Kogo, H. *et al.* HORMAD1¯dependent checkpoint/surveillance mechanism eliminates asynaptic oocytes. *Genes to Cells* 17, 439–454; 10.1111/j.1365-2443.2012.01600.x (2012).
7. Shin, Y.-H. *et al.* Hormad1 Mutation Disrupts Synaptonemal Complex Formation, Recombination, and Chromosome Segregation in Mammalian Meiosis. *PLoS Genetics* 6, e1001190; 10.1371/journal.pgen.1001190 (2010).
8. Turner, J. M. A. *et al.* Silencing of unsynapsed meiotic chromosomes in the mouse. *Nature Genetics* 37, 41–47; 10.1038/ng1484 (2004).
9. Turner, J. M. *et al.* BRCA1, Histone H2AX Phosphorylation, and Male Meiotic Sex Chromosome Inactivation. *Current Biology* 14, 2135–2142; 10.1016/j.cub.2004.11.032 (2004).
10. Mahadevaiah, S. K. *et al.* Extensive meiotic asynapsis in mice antagonises meiotic silencing of unsynapsed chromatin and consequently disrupts meiotic sex chromosome inactivation. *The Journal of Cell Biology* 182, 263–276; 10.1083/jcb.200710195 (2008).
11. Burgoyne, P., Mahadevaiah, S. K. & Turner, J. M. A. The consequences of asynapsis for mammalian meiosis. *Nature Reviews Genetics* 10, 207–216; 10.1038/nrg2505 (2009).
12. Royo, H. *et al.* ATR acts stage specifically to regulate multiple aspects of mammalian meiotic silencing. *Genes & development* 27, 1484–1494; 10.1101/gad.219477.113 (2013).
13. Ichijima, Y. *et al.* MDC1 directs chromosome-wide silencing of the sex chromosomes in male germ cells. *Genes & development* 25, 959–971; 10.1101/gad.2030811 (2011).
14. Jiao, X. *et al.* Aberrant activation of chromosome asynapsis checkpoint triggers oocyte elimination. *Nature Communications* 16, 2260; 10.1038/s41467-025-57702-z (2025).
15. Broering, T. J. *et al.* BRCA1 establishes DNA damage signaling and pericentric heterochromatin of the X chromosome in male meiosis. *The Journal of Cell Biology* 205, 663–675; 10.1083/jcb.201311050 (2014).

16. Bai, L. *et al.* BRCA1 safeguards genome integrity by activating chromosome asynapsis checkpoint to eliminate recombination-defective oocytes. *Proceedings of the National Academy of Sciences* 121, e2401386121; 10.1073/pnas.2401386121 (2024).

17. Ellnati, E. *et al.* DNA damage response protein TOPBP1 regulates X chromosome silencing in the mammalian germ line. *Proceedings of the National Academy of Sciences* 114, 12536–12541; 10.1073/pnas.1712530114 (2017).

18. Pereira, C. *et al.* Multiple 9-1-1 complexes promote homolog synapsis, DSB repair, and ATR signaling during mammalian meiosis. *eLife* 11; 10.7554/eLife.68677 (2022).

19. Turner, J. M., Mahadevaiah, S. K., Ellis, P. J., Mitchell, M. J. & Burgoyne, P. S. Pachytene Asynapsis Drives Meiotic Sex Chromosome Inactivation and Leads to Substantial Postmeiotic Repression in Spermatids. *Developmental Cell* 10, 521–529; 10.1016/j.devcel.2006.02.009 (2006).

20. Cloutier, J. M., Mahadevaiah, S. K., Ellnati, E., Tóth, A. & Turner, J. Mammalian meiotic silencing exhibits sexually dimorphic features. *Chromosoma* 125, 215–226; 10.1007/s00412-015-0568-z (2015).

21. Hirota, T. *et al.* SETDB1 Links the Meiotic DNA Damage Response to Sex Chromosome Silencing in Mice. *Developmental Cell* 47, 645-659.e6; 10.1016/j.devcel.2018.10.004 (2018).

22. Cloutier, J. M. *et al.* Histone H2AFX Links Meiotic Chromosome Asynapsis to Prophase I Oocyte Loss in Mammals. *PLoS Genetics* 11, e1005462; 10.1371/journal.pgen.1005462 (2015).

23. Abe, H. *et al.* Active DNA damage response signaling initiates and maintains meiotic sex chromosome inactivation. *Nature Communications* 13, 7212; 10.1038/s41467-022-34295-5 (2022).

24. Meng, E. C. *et al.* UCSF ChimeraX: Tools for structure building and analysis. *Protein Science* 32, e4792; 10.1002/pro.4792 (2023).

25. Abramson, J. *et al.* Accurate structure prediction of biomolecular interactions with AlphaFold 3. *Nature* 630, 493–500; 10.1038/s41586-024-07487-w (2024).

26. Kim, Y. *et al.* The Chromosome Axis Controls Meiotic Events through a Hierarchical Assembly of HORMA Domain Proteins. *Developmental Cell* 31, 487–502; 10.1016/j.devcel.2014.09.013 (2014).

27. Finsterbusch, F. *et al.* Alignment of Homologous Chromosomes and Effective Repair of Programmed DNA Double-Strand Breaks during Mouse Meiosis Require the Minichromosome Maintenance Domain Containing 2 (MCMDC2) Protein. *PLoS Genetics* 12, e1006393; 10.1371/journal.pgen.1006393 (2016).

28. Winters, T., McNicoll, F. & Jessberger, R. Meiotic cohesin STAG3 is required for chromosome axis formation and sister chromatid cohesion. *The EMBO Journal* 33, 1256–1270; 10.1002/embj.201387330 (2014).

29. Stanzione, M. *et al.* Meiotic DNA break formation requires the unsynapsed chromosome axis-binding protein IHO1 (CCDC36) in mice. *Nature Cell Biology* 18, 1208–1220; 10.1038/ncb3417 (2016).

30. Papanikos, F. *et al.* Mouse ANKRD31 Regulates Spatiotemporal Patterning of Meiotic Recombination Initiation and Ensures Recombination between X and Y Sex Chromosomes. *Molecular Cell* 74, 1069-1085.e11; 10.1016/j.molcel.2019.03.022 (2019).

31. Baudat, F., Manova, K., Yuen, J. P., Jasin, M. & Keeney, S. Chromosome Synapsis Defects and Sexually Dimorphic Meiotic Progression in Mice Lacking Spo11. *Molecular Cell* 6, 989–998; 10.1016/S1097-2765(00)00098-8 (2000).

32. Pittman, D. L. *et al.* Meiotic Prophase Arrest with Failure of Chromosome Synapsis in Mice Deficient for Dmc1 , a Germline-Specific RecA Homolog. *Molecular Cell* 1, 697–705; 10.1016/S1097-2765(00)80069-6 (1998).

33. Vries, F. A. T. de *et al.* Mouse Sycp1 functions in synaptonemal complex assembly, meiotic recombination, and XY body formation. *Genes & development* 19, 1376–1389; 10.1101/gad.329705 (2005).

34. Schneider, C. A., Rasband, W. S. & Eliceiri, K. W. NIH Image to ImageJ: 25 years of image analysis. *Nat Methods* 9, 671–675; 10.1038/nmeth.2089 (2012).

35. Schindelin, J. *et al.* Fiji: an open-source platform for biological-image analysis. *Nat Methods* 9, 676–682; 10.1038/nmeth.2019 (2012).

36. Zheng, G. X. Y. *et al.* Massively parallel digital transcriptional profiling of single cells. *Nature Communications* 8, 14049; 10.1038/ncomms14049 (2017).

37. R Core Team. *R: A Language and Environment for Statistical Computing* (R Foundation for Statistical Computing, Vienna, Austria, 2025).

38. Hao, Y. *et al.* Dictionary learning for integrative, multimodal and scalable single-cell analysis. *Nature Biotechnology* 42, 293–304; 10.1038/s41587-023-01767-y (2024).

39. Samuel Marsh, Maëlle Salmon, Paul Hoffman, kew24 & Mustafa Samet Pir. *samuel-marsh/scCustomize: Version 3.2.0* (Zenodo, 2025).

40. Wickham, H. *ggplot2. Elegant Graphics for Data Analysis.* 2nd ed. (Springer International Publishing; Imprint: Springer, Cham, 2016).

41. Kuznetsova, A., Brockhoff, P. B. & Christensen, R. H. B. lmerTest Package: Tests in Linear Mixed Effects Models. *J. Stat. Soft.* 82; 10.18637/jss.v082.i13 (2017).

42. Bates, D., Mächler, M., Bolker, B. & Walker, S. Fitting Linear Mixed-Effects Models Using lme4. *J. Stat. Soft.* 67; 10.18637/jss.v067.i01 (2015).

43. Montague, T. G., Cruz, J. M., Gagnon, J. A., Church, G. M. & Valen, E. CHOPCHOP: a CRISPR/Cas9 and TALEN web tool for genome editing. *Nucleic acids research* 42, W401-7; 10.1093/nar/gku410 (2014).

44. Labun, K., Montague, T. G., Gagnon, J. A., Thyme, S. B. & Valen, E. CHOPCHOP v2: a web tool for the next generation of CRISPR genome engineering. *Nucleic acids research* 44, W272-6; 10.1093/nar/gkw398 (2016).

45. O'Brien, A. & Bailey, T. L. GT-Scan: identifying unique genomic targets. *Bioinformatics* 30, 2673–2675; 10.1093/bioinformatics/btu354 (2014).
