# Supplementary material for: SYCP2 recruits HORMAD2 to chromosome axes for unsynapsed chromatin silencing and synapsis surveillance in meiosis
Source: Nat Commun. 2026 Jul 23;17:7122. doi: 10.1038/s41467-026-75839-3 (PMC13396342; doi:10.1038/s41467-026-75839-3)
Supplement: Supplementary file 2 — Description of Additional Supplementary Files [file 41467_2026_75839_MOESM2_ESM.pdf]

Description of Additional Supplementary Files

File Name: Supplementary Data 1

Description: Germline and somatic marker genes used for cluster identification.

Two sets of marker genes used to identify and separate germline and somatic cell populations in the single-cell RNA-seq data. Somatic markers (top section) were used to identify somatic cell-enriched clusters, which were excluded from further analysis. Germline markers (bottom section) were used to identify spermatogenic clusters and to perform supervised re-clustering of spermatogenic subpopulations.

File Name: Supplementary Data 2

Description: Summary of unsupervised clusters and marker-based cell-type annotations.

Clusters identified by unsupervised analysis of single-cell RNA-seq data and annotated based on expression of somatic and germline marker genes. Identified cell types include spermatogonia, spermatocytes, spermatids, Leydig cells, Sertoli cells, macrophages, and endothelial cells. Clusters with germline marker enrichment were subsequently selected for supervised re-clustering (see Supplementary Data 1). Key marker genes identifying dominant cell types in each cluster are listed.

File Name: Supplementary Data 3

Description: Summary of supervised clusters and marker-based cell-type annotations.

Clusters identified by supervised analysis of spermatogenic cells of single-cell RNA-seq data and annotated based on expression of germline marker genes. Key marker genes identifying dominant cell types in each cluster are listed.
