# Supplementary material for: SYCP2 recruits HORMAD2 to chromosome axes for unsynapsed chromatin silencing and synapsis surveillance in meiosis
Source: Nat Commun. 2026 Jul 23;17:7122. doi: 10.1038/s41467-026-75839-3 (PMC13396342; doi:10.1038/s41467-026-75839-3)
Supplement: Supplementary file 6 — Reporting Summary [file 41467_2026_75839_MOESM6_ESM.pdf]

nature portfolio

Corresponding author(s): Attila Tóth

Last updated by author(s): Jun 22, 2026

Reporting Summary

Nature Portfolio wishes to improve the reproducibility of the work that we publish. This form provides structure for consistency and transparency in reporting. For further information on Nature Portfolio policies, see our Editorial Policies and the Editorial Policy Checklist.

Statistics

For all statistical analyses, confirm that the following items are present in the figure legend, table legend, main text, or Methods section.

n/a	Confirmed	
☐	☒	The exact sample size (*n*) for each experimental group/condition, given as a discrete number and unit of measurement
☐	☒	A statement on whether measurements were taken from distinct samples or whether the same sample was measured repeatedly
☐	☒	The statistical test(s) used AND whether they are one- or two-sided<br>*Only common tests should be described solely by name; describe more complex techniques in the Methods section.*
☒	☐	A description of all covariates tested
☐	☒	A description of any assumptions or corrections, such as tests of normality and adjustment for multiple comparisons
☐	☒	A full description of the statistical parameters including central tendency (e.g. means) or other basic estimates (e.g. regression coefficient) AND variation (e.g. standard deviation) or associated estimates of uncertainty (e.g. confidence intervals)
☐	☒	For null hypothesis testing, the test statistic (e.g. *F*, *t*, *r*) with confidence intervals, effect sizes, degrees of freedom and *P* value noted<br>*Give P values as exact values whenever suitable.*
☒	☐	For Bayesian analysis, information on the choice of priors and Markov chain Monte Carlo settings
☒	☐	For hierarchical and complex designs, identification of the appropriate level for tests and full reporting of outcomes
☒	☐	Estimates of effect sizes (e.g. Cohen's *d*, Pearson's *r*), indicating how they were calculated

Our web collection on statistics for biologists contains articles on many of the points above.

Software and code

Policy information about availability of computer code

Data collection	Immunofluorescence images were collected using ZEN 2.3 Pro software (ZEISS). Single-cell RNA-seq data were generated using the 10x Genomics Chromium platform and sequenced on an Illumina platform. RT-qPCR data were collected using a CFX Opus 384 Real-Time PCR Detection System.
Data analysis	Immunofluorescence images were analyzed using Fiji/ImageJ version 1.54f and Adobe Photoshop CC19. Single-cell RNA-seq data were processed and analyzed using Cell Ranger version 6.1, Seurat version 5.0, and R version 4.4.1. RT-qPCR and other statistical analyses were performed using GraphPad Prism version 10 and R version 4.1.3. AlphaFold Server was used to generate protein structure models by AlphaFold 3, and models were visualized by the molecular graphics software ChimeraX version 1.7.1. Custom code for scRNA-seq data have been deposited in Zenodo [https://zenodo.org/records/18018240].

For manuscripts utilizing custom algorithms or software that are central to the research but not yet described in published literature, software must be made available to editors and reviewers. We strongly encourage code deposition in a community repository (e.g. GitHub). See the Nature Portfolio guidelines for submitting code & software for further information.

Data

Policy information about availability of data

All manuscripts must include a data availability statement. This statement should provide the following information, where applicable:

- Accession codes, unique identifiers, or web links for publicly available datasets
- A description of any restrictions on data availability
- For clinical datasets or third party data, please ensure that the statement adheres to our policy

The authors declare that all data supporting the findings of this study are available within the paper, its Supplementary Information, a source data file, and public data depositories. Data underlying figures has been deposited in the Biostudies database under accession number S-BSST1926 [https://www.ebi.ac.uk/biostudies/studies/S-BSST1926]. The single cell RNA-seq data generated in this study have been deposited in Array Express with accession number E-MTAB-16317 [https://www.ebi.ac.uk/biostudies/arrayexpress/studies/E-MTAB-16317]. Analyzed scRNA-seq data have been deposited in Zenodo [https://zenodo.org/records/18018240]. Source data are provided with this paper.

Research involving human participants, their data, or biological material

Policy information about studies with human participants or human data. See also policy information about sex, gender (identity/presentation), and sexual orientation and race, ethnicity and racism.

Reporting on sex and gender	Not relevant for this study.
Reporting on race, ethnicity, or other socially relevant groupings	Not relevant for this study.
Population characteristics	Not relevant for this study.
Recruitment	Not relevant for this study.
Ethics oversight	Not relevant for this study.

Note that full information on the approval of the study protocol must also be provided in the manuscript.

Field-specific reporting

Please select the one below that is the best fit for your research. If you are not sure, read the appropriate sections before making your selection.

☒ Life sciences ☐ Behavioural & social sciences ☐ Ecological, evolutionary & environmental sciences

For a reference copy of the document with all sections, see nature.com/documents/nr-reporting-summary-flat.pdf

Life sciences study design

All studies must disclose on these points even when the disclosure is negative.

Sample size	No formal sample-size calculations were performed. Nevertheless, sample sizes were chosen based on our past experiences and publications in the field to allow the detection of medium sized effects (equivalent to Cohen´s d 0.4-0.5) with confidence. Given that the examined mutant mice had very severe phenotypes in meiotic quality control, meiocyte apoptosis and fertility, the chosen sample sizes were appropriate and justified.<br>For examples of standards in the field, see the following papers: Wojtasz et. al. Meiotic DNA double-strand breaks and chromosome asynapsis in mice are monitored by distinct HORMAD2-independent and -dependent mechanisms. Genes Dev 26, 958-973 (2012), Stanzione, M. et al. Meiotic DNA break formation requires the unsynapsed chromosome axis-binding protein IHO1 (CCDC36) in mice. Nat Cell Biol 18, 1208–1220 (2016); Qiao, H. et al. Antagonistic roles of ubiquitin ligase HEI10 and SUMO ligase RNF212 regulate meiotic recombination. Nat Genet 46, 194–199 (2014); Zhang, J. et al. The BRCA2-MEILB2-BRME1 complex governs meiotic recombination and impairs the mitotic BRCA2-RAD51 function in cancer cells. Nat Commun 11, 2055 (2020); Holloway, J.K. et al. Mammalian CNTD1 is critical for meiotic crossover maturation and deselection of excess precrossover sites. J Cell Biol 205 (5), 633–641 (2014).
Data exclusions	No data were excluded from the analyses.
Replication	The reproducibility of all experimental findings was verified using independent biological replicates. All attempts at replication were successful, and results were reproducible across biological replicates. Conclusions were based on at least two, and in most cases three or more, biological replicates, with the number of replicates indicated in the figure legends. For comparisons within experiments, mice of different genotypes were littermates or were age matched to ensure comparability.
Randomization	Specific randomization methods are not relevant to the study. The study relies on comparison of wild type and various mutant mice that were generated by random segregation of alleles during sexual reproduction, which ensures random allocation of samples. Where control versus mutant mice were compared, samples were processed in parallel to eliminate batch effects.

Blinding

Blinding is not relevant to the study as, due to the drastic differences in phenotypes of wild type and mutant animals, even blinded investigator would be able to distinguish between the control and mutant samples. Yeast-two hybrid experiments are well controlled and have an unambiguous visual readout that is not influenced by the experimenter´s knowledge about sample identity. Images representing raw results are also presented in the figures of the manuscript, allowing direct evaluation by readers. Hence, blinding is not necessary.
All comparisons in yeast-two hybrid experiments were made between yeast transformed in the same experiment and grown on the same plate, so that the samples can be compared side-by-side without the need for blinding the investigator.

Reporting for specific materials, systems and methods

We require information from authors about some types of materials, experimental systems and methods used in many studies. Here, indicate whether each material, system or method listed is relevant to your study. If you are not sure if a list item applies to your research, read the appropriate section before selecting a response.

Materials & experimental systems

n/a	Involved in the study	
☐	☒	Antibodies
☒	☐	Eukaryotic cell lines
☒	☐	Palaeontology and archaeology
☐	☒	Animals and other organisms
☒	☐	Clinical data
☒	☐	Dual use research of concern
☒	☐	Plants

Methods

n/a	Involved in the study	
☒	☐	ChIP-seq
☒	☐	Flow cytometry
☒	☐	MRI-based neuroimaging

Antibodies

Antibodies used

Antibodies were used in the following applications:
IF: immunofluorescence
IP: immunoprecipitation
WB: immunoblotting/western blot

Primary antibodies used in this study are grouped below according to source.
Antibodies generated in this study:
rabbit anti-SYCP2 [IF 1:5000; IP 2 µg/reaction],
guinea pig anti-SYCP2 [IF 1:3000; WB 1:1000],
guinea pig anti-histone H1t [IF 1:2500],
rabbit anti-BRCA1 [IF 1:400],
rabbit anti-NOBOX [IF 1:2000],
guinea pig anti-DMC1 [IF 1:250].

Previously published non-commercial antibodies (previously described and validated in the cited publications):
chicken anti-SYCP3 [IF 1:800; WB 1:1000; https://doi.org/10.1371/journal.pgen.1006393],
mouse anti-SYCP3 [IF 1:2; https://doi.org/10.1002/embj.201387330],
chicken anti-SYCP1 [IF 1:600; https://doi.org/10.1016/j.molcel.2019.03.022],
chicken anti-IHO1 [IF 1:600; https://doi.org/10.1038/ncb3417],
rabbit anti-IHO1 [IF 1:2000; https://doi.org/10.1038/ncb3417],
guinea pig anti-HORMAD2 [IF 1:800 for nuclear spreads; IF 1:500 for cryosections; WB 1:2500; https://doi.org/10.1371/journal.pgen.1000702],
guinea pig anti-histone H1t [IF 1:20000; https://doi.org/10.1016/j.molcel.2019.03.022],
guinea pig anti-HORMAD1 [WB 1:3000; IP 2 µg/reaction; https://doi.org/10.1038/ncb2213].

Commercial antibodies and weblinks from manufacturers:
rabbit anti-SYCP1, Abcam, cat. no. ab15090, RRID: AB_301636 [IF 1:800; https://www.abcam.com/en-us/products/primary-antibodies/scp1-antibody-ab15090];
rabbit anti-RAD51, Abcam, cat. no. ab176458, RRID: AB_266540 [IF 1:800; https://www.abcam.com/en-us/products/primary-antibodies/rad51-antibody-ab176458];
mouse anti-DMC1 clone 2H12/4, Abcam, cat. no. ab11054, RRID: AB_297706 [IF 1:150; https://www.abcam.com/en-us/products/primary-antibodies/dmc1-antibody-2h12-4-ab11054];
rabbit anti-RPA32/RPA2 clone EPR2877Y, Abcam, cat. no. ab76420, RRID: AB_1524336 [IF 1:1000; https://www.abcam.com/en-us/products/primary-antibodies/rpa32-rpa2-antibody-epr2877y-ab76420];
mouse anti-phospho-Histone H2A.X Ser139 clone JBW301, Millipore, cat. no. 05-636, RRID: AB_309864 [IF 1:6000; https://www.merckmillipore.com/INTL/en/product/Anti-phospho-Histone-H2A.X-Ser139-Antibody-clone-JBW301,MM_NF-05-636];
goat anti-ATR N-19, Santa Cruz Biotechnology, cat. no. sc-1887, RRID: AB_630893 [IF 1:50; https://www.scbt.com/p/atr-antibody-n-19];
mouse anti-RNA polymerase II clone 8WG16, Santa Cruz Biotechnology, cat. no. sc-56767, RRID: AB_785522 [IF 1:50; https://www.scbt.com/p/pol-ii-antibody-8wg16];
rabbit anti-cleaved PARP Asp214, Cell Signaling Technology, cat. no. 9544, RRID: AB_2160724 [cryosection IF 1:250; https://www.cellsignal.com/products/primary-antibodies/cleaved-parp-asp214-antibody-mouse-specific/9544];

rabbit anti-histone H3, Abcam, cat. no. ab1791, RRID: AB_302613 [WB 1:200000; https://www.abcam.com/en-us/products/primary-antibodies/histone-h3-antibody-nuclear-marker-and-chip-grade-ab1791];
and mouse anti-GAPDH clone 6C5, Santa Cruz Biotechnology, cat. no. sc-32233, RRID: AB_627679 [WB 1:1000; https://datasheets.scbt.com/sc-32233.pdf],
mouse anti-SYCP3 clone Cor 10G11/7, Abcam, cat. no. ab97672, RRID: AB_10678841 [IF 1:200; https://www.abcam.com/en-us/products/primary-antibodies/scp3-antibody-cor-10g11-7-ab97672].

Secondary antibodies
Secondary antibodies used for western blotting were diluted 1:10000:
goat anti-rabbit IgG-HRP, Jackson ImmunoResearch, cat. no. 111-035-003, RRID: AB_2313567;
goat anti-guinea pig IgG-HRP, Jackson ImmunoResearch, cat. no. 706-035-148, RRID: AB_2340447;
goat anti-mouse IgG-HRP, Jackson ImmunoResearch, cat. no. 115-035-003, RRID: AB_10015289.

Secondary antibodies used for immunofluorescence were diluted 1:600 unless otherwise stated:
goat anti-rabbit IgG-AF405, Thermo Fisher Scientific, cat. no. A-31556, RRID: AB_221605;
goat anti-rabbit IgG-AF488, Thermo Fisher Scientific, cat. no. A-11034, RRID: AB_2576217;
goat anti-rabbit IgG-AF568, Thermo Fisher Scientific, cat. no. A-11036, RRID: AB_10563566;
goat anti-rabbit IgG-AF647, Thermo Fisher Scientific, cat. no. A-21244, RRID: AB_2535812;
highly cross-absorbed goat anti-rabbit IgG-AF647, Thermo Fisher Scientific, cat. no. A-21245, RRID: AB_2535813;
donkey anti-rabbit IgG-AF647, Jackson ImmunoResearch, cat. no. 711-495-152, RRID: AB_2315775;
donkey anti-rabbit IgG-DyLight 488, Jackson ImmunoResearch, cat. no. 711-485-152, RRID: AB_2492289 [1:300];
donkey anti-guinea pig IgG-DyLight 405, Jackson ImmunoResearch, cat. no. 706-475-148, RRID: AB_2340470;
goat anti-guinea pig IgG-AF488, Thermo Fisher Scientific, cat. no. A-11073, RRID: AB_2534117;
goat anti-guinea pig IgG-AF568, Thermo Fisher Scientific, cat. no. A-11075, RRID: AB_2534119;
goat anti-guinea pig IgG-AF647, Thermo Fisher Scientific, cat. no. A-21450, RRID: AB_2735091;
donkey anti-guinea pig IgG-AF647, Jackson ImmunoResearch, cat. no. 706-605-148, RRID: AB_2340476 [1:300];
donkey anti-guinea pig IgG-AF488, Jackson ImmunoResearch, cat. no. 706-545-148, RRID: AB_2340472;
goat anti-mouse IgG-AF405, Thermo Fisher Scientific, cat. no. A-31553, RRID: AB_221604 [1:200];
goat anti-mouse IgG-AF488, Thermo Fisher Scientific, cat. no. A-11029, RRID: AB_2534088;
goat anti-mouse IgG-AF568, Thermo Fisher Scientific, cat. no. A-11031, RRID: AB_144696;
donkey anti-mouse IgG-AF350, Thermo Fisher Scientific, cat. no. A-10035, RRID: AB_2534011 [1:300];
goat anti-chicken IgY-AF405, Abcam, cat. no. ab175675, RRID: AB_2810980;
goat anti-chicken IgY-AF488, Thermo Fisher Scientific, cat. no. A-11039, RRID: AB_2534096;
goat anti-chicken IgY-AF568, Thermo Fisher Scientific, cat. no. A-11041, RRID: AB_2534098;
goat anti-chicken IgY-AF647, Thermo Fisher Scientific, cat. no. A-21449, RRID: AB_2535866;
goat anti-rat IgG-AF488, Thermo Fisher Scientific, cat. no. A-11006, RRID: AB_2534074;
bovine anti-goat IgG-Rhodamine Red-X, Jackson ImmunoResearch, cat. no. 805-295-180, RRID: AB_2340881 [1:300].

Validation

Antibodies described and validated in previous studies were used as reported in the publications cited in the Methods and Supplementary Methods sections of the manuscript. The relevant DOIs are indicated in the “Antibodies used” section of the Reporting Summary.
Commercial antibodies were validated by the manufacturers for specificity and application as indicated on their websites; the relevant links are listed in the “Antibodies used” section of the Reporting Summary.
Antibodies generated in this study were validated by immunofluorescence, immunoblotting and/or IP combined with western blotting, demonstrating target-specific localization patterns and/or detection of proteins at the expected molecular weight. The same approaches were used to confirm specificity of commercial and previously published primary antibodies in the applications relevant to this study as listed for each antibody in the “Antibodies used” section of the reporting summary and the Methods section of the manuscript.

Animals and other research organisms

Policy information about studies involving animals; ARRIVE guidelines recommended for reporting animal research, and Sex and Gender in Research

Laboratory animals

Mice were kept on a C57BL/6J background (obtained from The Charles River Laboratories). Gonads were collected from mice after euthanasia. Experiments on spermatocytes and testes were carried out using samples from adult mice unless indicated otherwise. In the case of ovaries and oocytes, experiments were performed using 6-week-old and newborn mice. All animals were used and maintained in accordance with the German Animal Welfare legislation (“Tierschutzgesetz”). Mice were kept in a barrier facility in individually ventilated cages at 22–24 °C and 50–55% air humidity with a 14-h light/10-h dark cycle. Animals were fed a standard rat–mouse pellet diet. The maximum stocking density in type IIL cages was five mice per cage. Hygiene monitoring was carried out according to FELASA guidelines.

In addition to the newly generated Sycp2 mutant mice, previously published mutant mouse strains Hormad2, Dmc1, Iho1 (deletion), and Spo11 were used.

Wild animals

The study did not involve wild animals.

Reporting on sex

Both sexes were analyzed separately because male and female meiosis in mice take place at different developmental stages and are controlled by sex-specific mechanisms in addition to common pathways regulating mammalian meiosis. Male and female data were collected and reported separately.

Field-collected samples	The study did not involve samples collected from the field.
Ethics oversight	All mice were used and maintained in accordance with the German Animal Welfare legislation ("Tierschutzgesetz").All procedures pertaining to animal experiments were approved by the Governmental IACUC ("Landesdirektion Sachsen") and overseen by the animal ethics committee of the Technische Universität Dresden. The license number concerned with the present experiments with mice is TV A 8/2017 (DD24.1-5131/395/10).

Note that full information on the approval of the study protocol must also be provided in the manuscript.

Plants

Seed stocks	Not relevant for this study.
Novel plant genotypes	Not relevant for this study.
Authentication	Not relevant for this study.
