# Supplementary material for: SYCP2 recruits HORMAD2 to chromosome axes for unsynapsed chromatin silencing and synapsis surveillance in meiosis
Source: Nat Commun. 2026 Jul 23;17:7122. doi: 10.1038/s41467-026-75839-3 (PMC13396342; doi:10.1038/s41467-026-75839-3)
Supplement: Supplementary file 7 — Transparent Peer Review file [file 41467_2026_75839_MOESM7_ESM.pdf]

Peer Review File

SYCP2 recruits HORMAD2 to chromosome axes for unsynapsed chromatin silencing and synapsis surveillance in meiosis

Corresponding Author: Dr Attila Toth

This file contains all reviewer reports in order by version, followed by all author rebuttals in order by version.

Version 0:

Reviewer comments:

Reviewer #1

(Remarks to the Author)
"Chromosome axis protein SYCP2 recruits HORMAD2 to enable meiotic synapsis quality control in mice"
NCOMMS-25-103314

This paper describes the role of the closure motif of the Sycp2 protein in meiotic sex chromosome inactivation (MSCI) through the binding of a chromosome-axis protein, Hormad2, a meiosis-specific HORM domain protein in mouse meiosis. The authors created a mouse with a deletion of the CM motif (Exon 16 deletion) of SYCP2, which binds to the HORMAD domain of HORMAD2 (and-1), Sycp2^e16, and characterized the meiotic defects in details. They used immunostaining analysis of various marker proteins for meiotic recombination, chromosome synapsis, and MSCI (sex body formation) and nicely showed that the Sycp2^e16 spermatocytes lacked the cells after the mid-pachynema, thus are infertile. Importantly, the spermatocytes defective in the formation of the sex body, lacks the localization of HORMAD2, but not HORMAD1, to chromosome axes, resulting in reduced binding of checkpoint proteins, ATR and Brca1 (and gamma H2AX), on unpaired sex chromosomes for MSCI. In addition, the authors showed that Sycp2 CM is necessary for the elimination of oocytes defective in chromosome synapsis (not in meiotic recombination). These results nicely showed the critical role of the CM motif of SYCP2 in the recruitment of HORMAD2 proteins to the sex chromosomes. The experiments had been conducted with high quality, and most of the results in the paper support the authors' conclusion. The text is logically written and easy to read. There are several points which would be nicely addressed prior to publication.

Major points:
1. The length of the X chromosome in Sycp2 spermatocytes, shown in Fig. 5e and Supplementary Fig. 9, is longer than that in the control spermatocytes. Is this reproducible? Are similar defects seen in Hormad2 KO? If so, it should be mentioned in the Results section and discussed in the context of the chromosome axis compaction defect in the mutant.
2. A model shown in Figure 7e contains three question marks, which are irrelevant to the authors' main conclusion in the paper. It is more helpful to show a new model based on the results shown in the paper.

Minor points:
1. NO page numbers make it hard to point out.
2. The number of P-values in graphs (and also legends) in Figures and Supplementary Figures: For example, P=0.3808 in Fig. 3a. The numbers after the third decimal place are meaningless. Should be "0.38". Please think of valid numbers.
3. Throughout the text, words such as leptotene, zygotene, pachytene, and diplotene should be leptonema, zygonema, pachynema, and diplonema, respectively.
4. In the text, the authors classified stages of spermatocytes as stages I, II, III, etc. However, without the proper description of the stages in WT control. It is hard to follow the content.
5. It would be nice to move the AF3 predicted model of a complex of HORMAD2 with SYCP2-CM shown in the Supplement to the main Figure 1. Moreover, it would be nice to show the structure prediction of human (and other organisms) counterparts.
6. Line 148: It is important to mention that newly-constructed sycp2^e16 spermatocytes are different from Sypc2 knockout spermatocytes in meiotic defects.
7. Figure 5f, bottom right panel: A red line signal (Sycp3) denoted with "Y" looks quite long. Is the assignment of the Y

chromosomes OK in this figure?
8. Fig. 6d, e, and Fig. 6f, g showed the same data with different analyses. Either should be moved to the Supplementary Figure.

(Remarks on code availability)

Reviewer #2

(Remarks to the Author)
This study reports the in vivo role of the SYCP2 closure motif (CM) in the recruitment of HORMAD2 to the chromosomal axis in mice. SYCP2 is a component of the synaptonemal complex axial/lateral elements. Sycp2 exon 16 encoding the CM was deleted. The Sycp2e16 mutant males are sterile with meiotic arrest at the pachytene stage but females are fertile. In the Sycp2e16/e16 mutant, the SYCP2 protein lacking CM is present and localizes to the synaptonemal complex; HORMAD2 but not HORMAD1 is absent from the chromosomal axis, showing that SYCP2 is required for HORMAD2 recruitment but not HORMAD1. By making double mutants with Spo11 deficiency, the Sycp2e16 deletion, like Hormad2 deficiency, rescues the oocyte loss in the Spo11-deficient ovaries. HORMAD2 localizes to the XY body in pachytene spermatocytes. The Sycp2e16/e16 males, like Hormad2 knockout males, exhibit MSCI failure. This study shows that the Sycp2e16/e16 mice phenocopy the Hormad2-deficient mice. This study demonstrates the in vivo requirement of the SYCP2 CM in HORMAD2 recruitment and thus confirms the findings of previous biochemical and co-IP studies.

The study is well done. My main concerns are related to the accuracy of the conclusions, overemphasis on the importance of the current study, and underemphasis on the literature.

Major concerns:

1) Title: “Chromosome axis protein SYCP2 recruits HORMAD2 to enable meiotic synapsis quality control in mice”. This title only applies to females. In females, Sycp2e16 deletion leads to synapsis checkpoint failure in oocytes. This is not the case in Sycp2e16/e16 males, which display MSCI failure. These are related but distinct functions of HORMAD2. The majority of the experiments on Sycp2e16 mutants were done in males but the conclusions from males were not reflected in the title.

2) Abstract: “no mechanism for axis recruitment of HORMAD1 or HORMAD2 had been demonstrated” (Line 27). This is not correct. In West et al study (Elife. 2019 Jan 18:8:e40372. doi: 10.7554/eLife.40372), SYCP2 was shown to interact with HORMAD2 but not HORMAD1. In the West study, the CM in SYCP2 was identified and shown to be required for binding to HORMAD2. The current study is an in vivo study and confirms the findings in the eLife study. The previous findings in the eLife paper need to be described in more details in the Introduction.

3) Comparison of the meiotic phenotypes of Sycp2e16/e16 and Sycp2 null/Sycp2 coiled-coiled deletion mutants. Meiosis in Sycp2e16/e16 males progressed much further than in the other two Sycp2 mutants. It would be beneficial to describe the differences between these Sycp2 mutants in the Discussion.

(Remarks on code availability)
N/A

Reviewer #3

(Remarks to the Author)
This manuscript investigates the mechanism by which the chromosome axis protein SYCP2 recruits the HORMA domain protein HORMAD2 to unsynapsed chromosome axes, thereby enabling synapsis surveillance during mammalian meiosis. By generating a mouse line lacking the predicted closure motif (CM) of SYCP2, the authors demonstrate that this region is required explicitly for HORMAD2, but not HORMAD1, localization to the chromosome axes during the meiotic prophase. Loss of the CM abolishes SYCP2–HORMAD2 complex formation, prevents HORMAD2 loading onto axes, reduces ATR accumulation and signaling in unsynapsed regions, and results in checkpoint failure. This leads to the elimination of otherwise synapsis-proficient spermatocytes because of their inability to perform MSCI, and to the survival of asynaptic oocytes, because they fail to perform MSUC, phenocopying Hormad2-null mice. The study proposes that axial recruitment of HORMAD2 assembles a scaffold that enables ATR clustering and activation independently of DSBs, thereby linking axis architecture to synapsis quality control.
This is a high quality, thorough, and impressively comprehensive piece of work. The manuscript is very well written, with excellent logic in the experimental design and clear justification for each experimental step. While the level of detail may be somewhat extensive for a general readership (though entirely appropriate for meiosis specialists), the experiments are meticulously executed, and the data strongly support the manuscript's conclusions. Although this is a lengthy manuscript that requires the reader's full attention, the study will be of considerable interest not only to specialists in meiosis and reproductive biology, but also to researchers in cell biology, chromosome structure, and surveillance pathways.
I only have minor comments that could further improve clarity and accessibility of the manuscript:
• Line 32-34 - Regarding the sentence “Consequently, ATR accumulation and signaling on unsynapsed axes are reduced, and the prophase checkpoint malfunctions in a sexually dimorphic manner—causing aberrant elimination of synapsis-proficient spermatocytes and persistence of asynaptic oocytes”: As currently written, this sentence may be

confusing for readers who are not specialists in meiotic checkpoints. It may give the impression that the mutation itself causes different defects in males and females. However, the sexually dimorphic manifestations arise not because the mutation produces sex-specific defects, but because the meiotic checkpoint plays intrinsically different roles in spermatocytes versus oocytes. Thus, the observed dimorphism reflects the sex-specific function of the checkpoint, rather than the mutation behaving differently in each sex. I suggest clarifying this point to avoid misinterpretation by a broader audience.

• Line 109 - Please indicate the specific amino-acid residues that define the putative closure-motif (CM) region in SYCP2, so that readers can easily relate it to the mutation performed.

• Line 132 - Please provide a quantification of the cleaved PARP-positive spermatocytes in the different genotypes analyzed.

• Line 232 - I think it would be interesting for the authors to speculate in the discussion section how impaired MSCI could lead to illegitimate interactions between sex chromosomes and autosomes.

• Line 245 (Fig. 5A) – In the image provided for the Sycp2 CM-mutant spermatocytes, γH2AX appears to mainly coat an apparently well synapsed autosome. Was this common? Is this also applicable to other MSCI markers, such as BRCA1 or ATR? Is it also observed in Hormad2–/– spermatocytes?

• Line 263 (Fig. 5F-G) - Only two mice per genotype were used for the exclusion of the RNA pol II in the sex chromosomes analysis. Please justify the sample size and discuss limitations.

• Line 265 - As the authors are likely aware, scRNA-seq is not the optimal method for assessing transcriptional silencing of the XY chromosomes at pachynema. Although the results of this analysis are consistent with the other MSCI readouts (MSCI markers, RNA-polymerase exclusion, and qRT-PCR), I encourage the authors to consider corroborating the MSCI defects using RNA-FISH or another assay that directly detects nascent RNA, which is the standard used in the field for this kind of analysis.

• Line 308 - Although the data suggest that these mutants have no major defects in completing synapsis or progressing through meiotic prophase up to early pachytene stage, the authors should provide evidence that prophase staging is comparable across genotypes. Differences in staging or timing could otherwise confound the interpretation of the qRT-PCR results, since Zfy1 and Zfy2 are highly expressed at zygonema.

• Line 338 (Fig. 7A-B) - Even though it is common, there is a substantial variability in oocyte counts, especially for the WT and Sycp2 mutant mice. Please provide more details about how oocytes were quantified and discuss possible sources of variation.

• Line 458-461 (Discussion) - The sentence “Together, our data indicate that SYCP2 CM–mediated recruitment of HORMAD2 to unsynapsed axes enables efficient ATR activation. Reinforced by positive feedback loops within the ATR-activating network, this mechanism sustains high chromatin-bound ATR activity, which ensures elimination of asynaptic meiocytes—a key safeguard against aneuploidy in mammalian meiosis.” is inaccurate as written. In spermatocytes, this mechanism primarily enables silencing of the asynaptic XY chromosomal axes, not the elimination of the cells themselves. Therefore, the phrase “elimination of asynaptic meiocytes” is misleading in this context. I suggest replacing it with “silencing of asynaptic axes”, or alternatively, if the authors wish to retain the concept of cell elimination, then “meiocytes” should be replaced with “oocytes”, where ATR-dependent elimination indeed occurs.

• Figure 1d and Sup Fig 3e) — H1t signal appears weaker in Supplementary Fig. 3e compared to Fig. 1d. Consider adjusting intensity for consistency.

• Figure 2d-f - HORMAD2 appears as a double band in some gels (2f) but not others (2d,e). Please clarify the source of this difference.

• Supplementary Table 1 – Presenting fertility as “pups per breeding week” is an unusual and not particularly informative metric. It would be more useful to report standard reproductive indicators, such as total pups per female, average litter size, and/or number of litters per female.

(Remarks on code availability)

Reviewer #4

(Remarks to the Author)
I co-reviewed this manuscript with one of the reviewers who provided the listed reports. This is part of the Nature Communications initiative to facilitate training in peer review and to provide appropriate recognition for Early Career Researchers who co-review manuscripts.

(Remarks on code availability)

Version 1:

Reviewer comments:

Reviewer #1

(Remarks to the Author)
The authors nicely addressed the comments from the reviewers, including mine. This is a very nice paper.

(Remarks on code availability)

Reviewer #2

(Remarks to the Author)
My concerns have been adequately addressed in the revision.

(Remarks on code availability)
N/A

Reviewer #3

(Remarks to the Author)
The authors have satisfactorily addressed all of our comments and concerns through additional experiments, improved quantitative analyses, and careful revisions, and I have no further objections to the publication of this manuscript.

(Remarks on code availability)

Reviewer #4

(Remarks to the Author)
I co-reviewed this manuscript with one of the reviewers who provided the listed reports. This is part of the Nature Communications initiative to facilitate training in peer review and to provide appropriate recognition for Early Career Researchers who co-review manuscripts.

(Remarks on code availability)

Open Access This Peer Review File is licensed under a Creative Commons Attribution 4.0 International License, which permits use, sharing, adaptation, distribution and reproduction in any medium or format, as long as you give appropriate credit to the original author(s) and the source, provide a link to the Creative Commons license, and indicate if changes were made.
In cases where reviewers are anonymous, credit should be given to 'Anonymous Referee' and the source.
The images or other third party material in this Peer Review File are included in the article's Creative Commons license, unless indicated otherwise in a credit line to the material. If material is not included in the article's Creative Commons license and your intended use is not permitted by statutory regulation or exceeds the permitted use, you will need to obtain permission directly from the copyright holder.
To view a copy of this license, visit https://creativecommons.org/licenses/by/4.0/

Response to Reviewers

We thank the reviewers for their efforts and constructive suggestions. Below we provide point-by-point responses to the reviewers' comments on manuscript NCOMMS-25-103314. In our responses, we refer to line numbers in the revised manuscript, where we tracked changes; insertions are shown in red in the main text and deletions are shown in balloons. Because line numbers may change during reformatting, we either paste the revised text in the responses or, if the revised text is long, indicate the first words of newly inserted text in relevant responses to facilitate text searches.

Reviewer #1 (Remarks to the Author):

This paper describes the role of the closure motif of the Sycp2 protein in meiotic sex chromosome inactivation (MSCI) through the binding of a chromosome-axis protein, Hormad2, a meiosis-specific HORM domain protein in mouse meiosis. The authors created a mouse with a deletion of the CM motif (Exon 16 deletion) of SYCP2, which binds to the HORMAD domain of HORMAD2 (and-1), Sycp2^e16, and characterized the meiotic defects in details. They used immunostaining analysis of various marker proteins for meiotic recombination, chromosome synapsis, and MSCI (sex body formation) and nicely showed that the Sycp2^e16 spermatocytes lacked the cells after the mid-pachynema, thus are infertile. Importantly, the spermatocytes defective in the formation of the sex body, lacks the localization of HORMAD2, but not HORMAD1, to chromosome axes, resulting in reduced binding of checkpoint proteins, ATR and Brca1 (and gamma H2AX), on unpaired sex chromosomes for MSCI. In addition, the authors showed that Sycp2 CM is necessary for the elimination of oocytes defective in chromosome synapsis (not in meiotic recombination). These results nicely showed the critical role of the CM motif of SYCP2 in the recruitment of HORMAD2 proteins to the sex chromosomes. The experiments had been conducted with high quality, and most of the results in the paper support the authors' conclusion. The text is logically written and easy to read. There are several points which would be nicely addressed prior to publication.

Response:

We thank the reviewer for the positive evaluation of our manuscript and for recognizing the relevance of our findings to the meiosis field. We also appreciate the thoughtful comments and suggestions provided below.

Major points:

Comment 1: 1. The length of the X chromosome in Sycp2 spermatocytes, shown in Fig. 5e and Supplementary Fig. 9, is longer than that in the control spermatocytes. Is this reproducible? Are

similar defects seen in Hormad2 KO? If so, it should be mentioned in the Results section and discussed in the context of the chromosome axis compaction defect in the mutant.

Response:

Defects in sex chromosome compaction are a recognized feature of mutations that disrupt XY body formation and silencing (Ichijima et al., 2011; ref. [10]; Hirota et al., 2018; ref. [17]). As requested by the reviewer, we assessed sex chromosome compaction in *Sycp2*$^{e16/e16}$ and *Hormad2*$^{-/-}$ spermatocytes. We quantified the X–Y end-to-end distance normalized to the square root of the nuclear area, to account for differences in nuclear spreading. Both mutants consistently exhibited increased X–Y end-to-end distances across biological replicates compared with WT, indicative of a more extended XY configuration, which is shown in Supplementary Fig. 9d and mentioned in the revised text (see lines 306-310).

The newly inserted paragraph reads:

" Consistent with defective sex body formation, both *Sycp2*$^{e16/e16}$ and *Hormad2*$^{-/-}$ spermatocytes exhibited impaired sex chromosome compaction, reflected by an increased X–Y end-to-end distance and a more extended XY configuration (Fig. 5a, c, and e and Supplementary Fig. 9d). Similar impairment of sex chromosome compaction has been reported in other mutants with defective sex body formation and silencing [10,17]."

A corresponding method section is inserted at lines 962-968 (start with: "Quantification of sex chromosome compaction…").

Comment 2: 2. A model shown in Figure 7e contains three question marks, which are irrelevant to the authors' main conclusion in the paper. It is more helpful to show a new model based on the results shown in the paper.

Response:

We appreciate this point. Our intention in including the question marks in the original model was to highlight aspects that remain unresolved in the field and are relevant to the context of our study. Nevertheless, we agree with the reviewer that the use of multiple question marks distracted from the main message of the manuscript. Hence, we revised the model to more directly reflect our findings by separately depicting the wild-type (WT) and SYCP2 closure motif–deficient mutant conditions. Due to space constraints, the revised model is now presented as a newly prepared Fig. 8.

Comment 3: Minor points: 1. NO page numbers make it hard to point out.

Response:

We have added page numbers throughout the document to make specific sections easier to reference.

Comment 4: Minor points: 2. The number of P-values in graphs (and also legends) in Figures and Supplementary Figures: For example, P=0.3808 in Fig. 3a. The numbers after the third decimal place are meaningless. Should be "0.38". Please think of valid numbers.

Response:

As requested, we revisited the presentation of P values throughout the figures and legends. P values were calculated by GraphPad Prism or R, which report exact P values to different numbers of decimal places. We agree that excessively long decimal places reduce readability and should be avoided, yet reporting P values to four decimal places reflects the default output format of GraphPad Prism for P values ≥ 0.0001. Nevertheless, to improve readability and consistency, we revised the display format as follows: P values ≥ 0.001 are reported to three decimal places; P values between 0.001 and 0.0001 are reported to four decimal places, in line with GraphPad Prism default output; and P values < 0.0001 calculated by R are reported in scientific notation with up to two significant figures (e.g., 1.2e-7) where applicable; in cases where the statistical output provides fewer significant figures (e.g., 6e-21), we retained that format. These changes affect presentation only; the underlying statistical tests, conclusions, and exact P values in the source file are unchanged.

Comment 5: Minor points: 3. Throughout the text, words such as leptotene, zygotene, pachytene, and diplotene should be leptonema, zygonema, pachynema, and diplonema, respectively.

Response:

We appreciate the reviewer's point. However, leptotene, zygotene, pachytene, and diplotene are equally correct synonyms of leptonema, zygonema, pachynema, and diplonema, both sets of terms being derived from the same Greek roots with identical meaning. Both terminologies have been used interchangeably in the meiosis literature for decades, and leptotene, zygotene, pachytene, and diplotene remain widely used, including in recent Nature Communications publications. As these terms have been applied consistently throughout our manuscript, we respectfully prefer to retain them for the sake of consistency and readability.

Comment 6: Minor points: 4. In the text, the authors classified stages of spermatocytes as stages I, II, III, etc. However, without the proper description of the stages in WT control. It is hard to follow the content.

Response:

We appreciate the reviewer's comment; however, there appears to be a misunderstanding regarding our staging nomenclature. The Roman numerals in our manuscript refer exclusively to stages of the seminiferous epithelial cycle, not to spermatocyte developmental stages. As noted in our original submission, the criteria used to define these epithelial cycle stages are described in detail in the Methods section, and both the main text (line 160, stating "see 'Methods' for epithelial cycle staging [49]") and the legend for Fig. 1 direct readers to this description upon first mention.

Nevertheless, this comment prompted us to review that section, and we noticed that our original description of tubule stage IV was pooled with stages I–III. Because the analysis of stage IV seminiferous tubules is a central part of our manuscript, we have expanded the relevant Methods section (lines 863-948, start with "Staging of mouse seminiferous tubule cross sections….") to explicitly define the distinguishing features of stage IV tubules. We hope this clarification resolves the concern.

Comment 7: Minor points: 5. It would be nice to move the AF3 predicted model of a complex of HORMAD2 with SYCP2-CM shown in the Supplement to the main Figure 1. Moreover, it would be nice to show the structure prediction of human (and other organisms) counterparts.

Response:

In response to the reviewer's suggestion, we have now included the predicted complex of human SYCP2 and HORMAD2 in the same supplementary figure (Supplementary Fig. 2), alongside the original models. An AF3 model prediction for a potential complex of human SYCP2 and HORMAD1 was published previously (Jiao et al. 2025; ref. [29], https://doi.org/10.1038/s41467-025-57702-z), and was therefore not included in the figure. We mention and reference this previous AF3 prediction in the introduction of the revised manuscript (line 131-133, starting with "However, AlphaFold 3 modeling and …"). We hope that these additions address the reviewer's request. We chose to retain these structural predictions in the Supplementary Information to preserve the focus and clarity of Fig. 1.

Prior *in vitro* protein interaction and protein structure studies have predicted HORMAD protein interactions that may underlie HORMAD assembly on chromosome axes in diverse taxa. One of the main points of this manuscript is testing the *in vivo* relevance of previously predicted HORMAD complexes with the axis scaffold protein SYCP2 in mice, showing that the predicted SYCP2 CM is

required only for HORMAD2 but not HORMAD1 functions in mice, despite AF3 predictions suggesting otherwise. We therefore think that adding further *in silico* predictions for additional taxa would distract from the main aims, go beyond the scope of the current manuscript, and be redundant with prior *in vitro* studies. Therefore, we have chosen not to expand on further AF3 predictions that are not directly related to the current message of the manuscript.

Comment 8: Minor points: 6. Line 148: It is important to mention that newly-constructed sycp2^e16 spermatocytes are different from Sypc2 knockout spermatocytes in meiotic defects.

Response:

We agree that briefly comparing *Sycp2*$^{e16/e16}$ phenotypes with the phenotypes of previously reported *Sycp2* mutant lines will help readers interpreting the manuscript. Therefore, we added a sentence in the Results (lines 176-181) and a sentence in the Discussion (line 455-457), explaining that the largely normal axis assembly and recombination in *Sycp2*$^{e16/e16}$ meiocytes contrast with severely disrupted axis assembly and defective recombination in mutants that lack the entire SYCP2 or its C-terminal coiled-coil domain, which mediates SYCP3 binding. These revisions make the distinction between the phenotypes of *Sycp2*$^{e16/e16}$ and other reported *Sycp2* mutants clearer, addressing both reviewer #1 comment 8 and reviewer #2 comments 3.

The newly inserted paragraph in the Results:

“These phenotypes contrasted with the severely disrupted axis assembly and synapsis in mutants that lack the entire SYCP2 or its C-terminal coiled-coil domain, which mediates SYCP3 binding[43,46,50]. Thus, unlike the SYCP3-interacting regions of SYCP2, the SYCP2 CM was not required for axis assembly or for the scaffolding function of axes in SC formation, which allowed direct comparison of prophase stages in WT and *Sycp2*$^{e16/e16}$ spermatocytes based on axis morphology.”

The newly inserted paragraph in the Discussion:

“Unlike SYCP2-null or SYCP2 coiled-coil–deficient mutants, *Sycp2*$^{e16/e16}$ meiocytes do not display severe axis and SC assembly failure or associated recombination defects[43,46,50].”

Comment 9: Minor points: 7. Figure 5f, bottom right panel: A red line signal (Sycp3) denoted with “Y” looks quite long. Is the assignment of the Y chromosomes OK in this figure?

Response:

Yes, the chromosome assignment in this panel is correct. X and Y chromosomes are less compacted in *Sycp2*$^{e16/e16}$ spermatocytes than in WT, as shown by our measurements in Supplementary Fig. 9d, which often result in an overlap or entanglement between autosomal axes and X and Y axes. In this image, both the Y axis, located to the right of the PAR region, and the X axis, stretching to the left of the PAR, overlap with autosomal axes. To avoid possible confusion, we labelled the PAR region in Fig. 5f and adjusted the Y chromosome label to point unambiguously to the unsynapsed Y axis rather than to the region where it overlaps with an autosome.

Comment 10: Minor points: 8. Fig. 6d, e, and Fig. 6f, g showed the same data with different analyses. Either should be moved to the Supplementary Figure.

Response:

We agree that all the panels mentioned support the key conclusion that *Zfy1* and *Zfy2* expression is elevated in *Sycp2*$^{e16/e16}$ spermatocytes. However, the figures do not present the same data: Fig. 6d, e show RT–qPCR analysis of total testis samples, whereas Fig. 6f, g show single-cell RNA-seq analyses across zygotene and pachytene spermatocytes. These distinct analytical approaches provide complementary evidence for a critically important conclusion in the manuscript, each with distinct strengths. We have therefore decided to retain both panels in the main figure.

Reviewer #2 (Remarks to the Author):

This study reports the in vivo role of the SYCP2 closure motif (CM) in the recruitment of HORMAD2 to the chromosomal axis in mice. SYCP2 is a component of the synaptonemal complex axial/lateral elements. Sycp2 exon 16 encoding the CM was deleted. The Sycp2e16 mutant males are sterile with meiotic arrest at the pachytene stage but females are fertile. In the Sycp2e16/e16 mutant, the SYCP2 protein lacking CM is present and localizes to the synaptonemal complex; HORMAD2 but not HORMAD1 is absent from the chromosomal axis, showing that SYCP2 is required for HORMAD2 recruitment but not HORMAD1. By making double mutants with Spo11 deficiency, the Sycp2e16 deletion, like Hormad2 deficiency, rescues the oocyte loss in the Spo11-deficient ovaries. HORMAD2 localizes to the XY body in pachytene spermatocytes. The Sycp2e16/e16 males, like Hormad2 knockout males, exhibit MSCI failure. This study shows that the Sycp2e16/e16 mice phenocopy the Hormad2-deficient mice. This study demonstrates the in vivo requirement of the SYCP2 CM in HORMAD2 recruitment and thus confirms the findings of previous biochemical and co-IP studies.

The study is well done. My main concerns are related to the accuracy of the conclusions, overemphasis on the importance of the current study, and underemphasis on the literature.

Response:

We thank the reviewer for the careful evaluation of our manuscript, for recognizing the importance of the study, and for the constructive suggestions.

Major concerns:

Comment 1: 1) Title: “Chromosome axis protein SYCP2 recruits HORMAD2 to enable meiotic synapsis quality control in mice”. This title only applies to females. In females, Sycp2e16 deletion leads to synapsis checkpoint failure in oocytes. This is not the case in Sycp2e16/e16 males, which display MSCI failure. These are related but distinct functions of HORMAD2. The majority of the experiments on Sycp2e16 mutants were done in males but the conclusions from males were not reflected in the title.

Response:

We agree that the female synapsis-checkpoint phenotype (persistence of asynaptic oocytes in DSB-deficient settings) cannot be equated with the male meiotic sex chromosome inactivation (MSCI) phenotype. We also agree that our previous title could be interpreted as referring primarily to females and therefore did not adequately encompass the male phenotype.

Our data identify SYCP2-mediated recruitment of HORMAD2 to chromosome axes as a key requirement for axis-associated ATR signaling in both sexes. This ATR signaling promotes meiotic silencing of unsynapsed chromatin (MSUC), which underlies MSCI in spermatocytes, and it supports synapsis-checkpoint–triggered loss of asynaptic oocytes. To comprehensively reflect the breadth of these functions, we have changed the title to: “**SYCP2 recruits HORMAD2 for chromosome-axis-associated ATR signaling, unsynapsed chromatin silencing, and synapsis surveillance in meiosis**”.

Comment 2: 2) Abstract: “no mechanism for axis recruitment of HORMAD1 or HORMAD2 had been demonstrated” (Line 27). This is not correct. In West et al study (Elife. 2019 Jan 18:8:e40372. doi: 10.7554/eLife.40372), SYCP2 was shown to interact with HORMAD2 but not HORMAD1. In the West study, the CM in SYCP2 was identified and shown to be required for binding to HORMAD2. The current study is an in vivo study and confirms the findings in the eLife study. The previous findings in the eLife paper need to be described in more details in the Introduction.

Response:

We thank the reviewer for this important point. We agree that our original Abstract wording did not sufficiently distinguish between the lack of *in vivo* functional data and the existing

biochemical/exogenous interaction data. We also agree that West et al. (ref. [43]) provided relevant *in vitro* protein interaction data. Accordingly, we discussed West et al. (ref. [43]) in the Introduction of the original manuscript (first cited in a sentence in lines 120-123, sentence starts with "The ligand is then encircled…"), describing the identification of a closure motif (CM) in SYCP2 and its interaction with HORMAD proteins in *in vitro*/exogenous assays.

Importantly, while suggestive, demonstrating HORMAD binding in the previously published protein interaction assays does not establish an *in vivo* mechanism of chromosome-axis recruitment for HORMADs, the *in vivo* selectivity of CM binding (HORMAD2 vs. HORMAD1), or the functional relevance of HORMAD interactions for ATR signaling and checkpoint function. In particular, the *in vivo* specificity of CM binding remained uncertain, because West et al. (ref. [43]) noted technical limitations in testing HORMAD1. In addition, AlphaFold 3 modeling and interaction data in exogenous mammalian cell-based assays (Jiao et al., 2025; ref. [29]) suggest that the SYCP2 CM also interacts with HORMAD1. Adding to this complexity, cohesins were implicated in axial HORMAD recruitment (Fujiwara et al., 2020; ref. [46]), and interactions of HORMA domains and C-terminal closure motifs in both HORMAD1 and HORMAD2 were suggested to contribute to their axial assembly (Kim et al., 2014; ref. [45], as noted in lines 124-130, relevant sentences starting with "Interactions of meiotic HORMA domain…"). Thus, prior studies left open whether the SYCP2 CM functions *in vivo* as a recruiter of HORMADs, whether it acts selectively on HORMAD2 versus HORMAD1, and whether it is important for their known functions *in vivo*.

Our *in vivo* genetics answers these key unresolved questions by establishing the *in vivo* requirement and selectivity of the SYCP2 closure motif, which could not be inferred unambiguously from prior *in vitro*/exogenous interaction assays. We have therefore revised the Abstract to explicitly distinguish prior interaction data from an *in vivo* recruitment mechanism, and expanded the relevant Introduction text to more clearly summarize the prior evidence from West et al. (ref. [43]) and other studies while highlighting the need for an *in vivo* genetic test.

The relevant revised Abstract text reads (lines 26-48):

"However, the *in vivo* mechanism for axial HORMAD1 and HORMAD2 recruitment and its relevance to checkpoint signaling had not been established, although the chromosome-axis component SYCP2 was proposed to contain a candidate HORMAD-binding closure motif (CM). We show that deletion of the SYCP2 CM disrupts SYCP2–HORMAD2 complexes and selectively prevents HORMAD2 axis binding without affecting axis assembly, recombination, or axial HORMAD1 recruitment."

The relevant additions to the Introduction read (lines 130-135):

“In particular, preferential interaction of the SYCP2 CM with HORMAD2 was reported in *in vitro* assays [43], with the authors noting technical limitations for testing HORMAD1. However, AlphaFold 3 modeling and mammalian cell-based interaction data have suggested that the SYCP2 CM may also interact with HORMAD1 [29]. Nevertheless, the *in vivo* roles of these interactions have not been reported, and the mechanism of HORMAD1 and HORMAD2 recruitment to chromosomes remains unclear.”

This wording preserves the key message of our original statement while more explicitly acknowledging the prior interaction work.

Comment 3: 3) Comparison of the meiotic phenotypes of Sycp2e16/e16 and Sycp2 null/Sycp2 coiled-coiled deletion mutants. Meiosis in Sycp2e16/e16 males progressed much further than in the other two Sycp2 mutants. It would be beneficial to describe the differences between these Sycp2 mutants in the Discussion.

Response:

We have now addressed this point and the related comment 8 of reviewer #1 in both the relevant Results section (lines 176–181, the first sentence starting with “These phenotypes contrasted…”) and the Discussion (lines 455-457, sentence starting with “Unlike SYCP2-null or…”). Specifically, we added text comparing the meiotic phenotype of Sycp2$^{e16/e16}$ spermatocytes with those previously reported for *Sycp2*-null and *Sycp2* coiled-coil deletion mutants, clarifying that the *Sycp2$^{e16/e16}$* phenotype is distinct from and less severe than those of the previously described *Sycp2* mutants.

Reviewer #3 (Remarks to the Author):

This manuscript investigates the mechanism by which the chromosome axis protein SYCP2 recruits the HORMA domain protein HORMAD2 to unsynapsed chromosome axes, thereby enabling synapsis surveillance during mammalian meiosis. By generating a mouse line lacking the predicted closure motif (CM) of SYCP2, the authors demonstrate that this region is required explicitly for HORMAD2, but not HORMAD1, localization to the chromosome axes during the meiotic prophase. Loss of the CM abolishes SYCP2–HORMAD2 complex formation, prevents HORMAD2 loading onto axes, reduces ATR accumulation and signaling in unsynapsed regions, and results in checkpoint failure. This leads to the elimination of otherwise synapsis-proficient spermatocytes because of their inability to perform MSCI, and to the survival of asynaptic oocytes, because they fail to perform MSUC, phenocopying Hormad2-null mice. The study proposes that axial recruitment of HORMAD2 assembles a scaffold that enables ATR clustering and activation

independently of DSBs, thereby linking axis architecture to synapsis quality control.
This is a high quality, thorough, and impressively comprehensive piece of work. The manuscript is very well written, with excellent logic in the experimental design and clear justification for each experimental step. While the level of detail may be somewhat extensive for a general readership (though entirely appropriate for meiosis specialists), the experiments are meticulously executed, and the data strongly support the manuscript's conclusions. Although this is a lengthy manuscript that requires the reader's full attention, the study will be of considerable interest not only to specialists in meiosis and reproductive biology, but also to researchers in cell biology, chromosome structure, and surveillance pathways.

Response:

We thank the reviewer for the positive evaluation of our manuscript, for recognizing its contribution to the field, and for the constructive suggestions for improvement.

Detailed reviewer points:

I only have minor comments that could further improve clarity and accessibility of the manuscript:

Comment 1: • Line 32-34 - Regarding the sentence “Consequently, ATR accumulation and signaling on unsynapsed axes are reduced, and the prophase checkpoint malfunctions in a sexually dimorphic manner—causing aberrant elimination of synapsis-proficient spermatocytes and persistence of asynaptic oocytes”: As currently written, this sentence may be confusing for readers who are not specialists in meiotic checkpoints. It may give the impression that the mutation itself causes different defects in males and females. However, the sexually dimorphic manifestations arise not because the mutation produces sex-specific defects, but because the meiotic checkpoint plays intrinsically different roles in spermatocytes versus oocytes. Thus, the observed dimorphism reflects the sex-specific function of the checkpoint, rather than the mutation behaving differently in each sex. I suggest clarifying this point to avoid misinterpretation by a broader audience.

Response:

We revised the sentence (now lines 48-52) to emphasize that sexual dimorphism of the checkpoint mechanisms underlies the distinct phenotypes of SYCP2 CM loss in males and females:

“Consequently, ATR accumulation and signaling on unsynapsed axes are reduced, and the prophase checkpoint malfunctions, manifesting in aberrant elimination of synapsis-proficient spermatocytes and persistence of asynaptic oocytes, which reflect sex-specific characteristics of checkpoint mechanisms.”

Comment 2: • Line 109 - Please indicate the specific amino-acid residues that define the putative closure-motif (CM) region in SYCP2, so that readers can easily relate it to the mutation performed.

Response:

This information was included in the first sentence of the Results section of the manuscript. Specifically, in lines 137-141 we indicate that the postulated CM in SYCP2 corresponds to residues 395–414, and that this region was removed in mice by deletion of exon 16 of *Sycp2*, which encodes residues 395–419. The relevant sentence starts with “To investigate the mechanism and…”.

Comment 3: • Line 132 - Please provide a quantification of the cleaved PARP-positive spermatocytes in the different genotypes analyzed.

Response:

In the initially submitted version of the manuscript, we reported that *Sycp2*$^{e16/e16}$ mice showed an absence of post-meiotic spermatogenic cells and strongly H1t-positive spermatocytes together with elevated cleaved PARP staining at stage IV of the seminiferous epithelial cycle. These observations collectively indicated a spermatogenic block at mid-pachytene. As requested, we have now quantified apoptosis by scoring stage IV seminiferous tubules containing cleaved PARP-positive spermatocytes in cross-sections from WT and *Sycp2*$^{e16/e16}$ testes across three (WT) and two (*Sycp2*$^{e16/e16}$) biological replicates. We found that 44 out of 56 (79%) of stage IV tubules in Sycp2$^{e16/e16}$ testes contained cleaved PARP-positive cells, compared with only 1 out of 38 (3%) in WT. These results unambiguously support the original phenotype description and are reported in lines 161-165 of the revised manuscript and Supplementary Table 3. The revised sentence in the Results reads: “Correspondingly, detection of the apoptosis marker cleaved PARP revealed high levels of spermatocyte death at stage IV of the seminiferous epithelial cycle (Fig. 1d), with 79% (44 out of 56) of stage IV seminiferous tubules showing apoptotic cells in *Sycp2*$^{e16/e16}$ testes, compared to 3% (1 out of 38) in WT testes across two (*Sycp2*$^{e16/e16}$) and three (WT) biological replicates (Supplementary Table 3.).”

Comment 4: • Line 245 (Fig. 5A) – In the image provided for the Sycp2 CM-mutant spermatocytes, γH2AX appears to mainly coat an apparently well synapsed autosome. Was this common? Is this also applicable to other MSCI markers, such as BRCA1 or ATR? Is it also observed in Hormad2−/− spermatocytes?

Response:

Our original counts of abnormally formed γH2AX-rich domains (83.7% of spermatocytes in both genotypes) included cells where γH2AX-rich chromatin domains largely overlapped with synapsed autosomes. To address the questions of the reviewer we have now performed additional analysis on these cells, revealing that only 3.1% of Sycp2$^{e16/e16}$ spermatocytes and 2.85% of *Hormad2*$^{-/-}$ spermatocytes exhibited γH2AX-rich chromatin domains that largely overlapped with synapsed autosomes. These quantifications are consistent with prior analysis of *Hormad2*$^{-/-}$ meiocytes (Wojtasz et al., 2012; ref. [35]). We further examined BRCA1 in *Sycp2*$^{e16/e16}$ spermatocytes and found that none of these autosome-overlapping γH2AX domains associated with BRCA1 accumulation on synapsed autosomal axes, raising the possibility that the autosome-associated γH2AX domains may represent abnormally stretched out/uncondensed sex chromosomal chromatin instead of true ectopic activation of ATR signals on synapsed autosomes.

To clarify the phenotype, we have now included additional description of the γH2AX patterns in the revised text (lines 285-288.), which reads:" Among these, 3.1% of *Sycp2*$^{e16/e16}$ spermatocytes and 2.85% of *Hormad2*$^{-/-}$ spermatocytes showed γH2AX-rich chromatin domains that largely overlapped with synapsed autosomes. However, BRCA1 was not detected on the same synapsed autosomes (n = 20 cells from 2 mice)."

Comment 5: • Line 263 (Fig. 5F-G) - Only two mice per genotype were used for the exclusion of the RNA pol II in the sex chromosomes analysis. Please justify the sample size and discuss limitations.

Response:

Due to unambiguous effects, only two experimental replicates were quantified originally, which indicated that the results were reproducible. To reinforce this conclusion and address the reviewer´s concern, we have now added a third biological replicate, which reproduced the result from the first two replicates. Fig. 5g has been updated accordingly. Thus, across all three biological replicates, RNA Pol II was consistently detected on the sex chromosomes in *Sycp2*$^{e16/e16}$ spermatocytes, indicating defective MSCI in the mutant.

Comment 6: • Line 265 - As the authors are likely aware, scRNA-seq is not the optimal method for assessing transcriptional silencing of the XY chromosomes at pachynema. Although the results of this analysis are consistent with the other MSCI readouts (MSCI markers, RNA-polymerase exclusion, and qRT-PCR), I encourage the authors to consider corroborating the MSCI defects

using RNA-FISH or another assay that directly detects nascent RNA, which is the standard used in the field for this kind of analysis.

Response:

We agree that RNA-FISH is one of several methods that have been used in various valid combinations to evaluate MSCI in the field. However, we respectfully note that recent studies (e.g., Ernst et al., 2019; ref. [60]; DOI: 10.1038/s41467-019-09182-1; Lau et al., 2020; DOI: 10.1016/j.devcel.2020.07.018) have demonstrated that scRNA-seq is a powerful and reliable method for assaying MSCI in mice and other organisms, combining several strengths of complementary approaches. In particular, scRNA-seq (i) provides quantitative readouts of X/Y transcription in large numbers of individual cells whose prophase stage can be assigned using autosome-linked meiotic marker genes, (ii) provides chromosome-wide data alongside gene-level information, (iii) allows comparison of sex-chromosomal transcription to autosomal transcription as an internal control for MSCI assessment within each cell, and (iv) prevents confounding from altered testis cellular composition in mutants by comparing stage-matched single-cell populations. Therefore, we consider scRNA-seq to be a well-suited and informative method for assessing MSCI, giving high confidence in conclusions about the efficiency of sex-chromosomal silencing in the mutants compared to WT during pachytene.

Furthermore, we note that in the present study, our conclusion that sex body formation and MSCI are impaired in *Sycp2*$^{e16/e16}$ spermatocytes does not rely on scRNA-seq alone. Rather, it is supported by multiple independent lines of evidence. First, we observe defective sex body formation, including abnormal γH2AX domain formation and reduced, discontinuous BRCA1 and ATR accumulation on unsynapsed sex-chromosome axes. Second, RNA polymerase II is not properly excluded from sex chromosomes in mutant pachytene spermatocytes, consistent with defective silencing. Notably, quantification of RNA Pol II exclusion from the sex body, like RNA-FISH, provides a conclusive cytological readout of transcriptional silencing on the sex chromosomes, and is a well-established assay for MSCI failure. Third, RT-qPCR shows increased expression of the MSCI-sensitive Y-linked genes *Zfy1* and *Zfy2* in testes at a developmental stage where a major spermatogenic cell population is in early pachytene. In the revised manuscript, we also expanded this RT-qPCR analysis showing that autosomal transcripts that mark early prophase do not change their abundance, which strongly indicates that altered *Zfy1* and *Zfy2* transcript levels reflect defects in sex chromosome silencing rather than altered cellular composition in the analyzed *Sycp2*$^{e16/e16}$ testes (see also response to reviewer 3 comment 7). Finally, our scRNA-seq analysis demonstrates a broader failure to fully suppress X- and Y-linked transcription in early-to-

mid pachytene cells in addition to confirming elevated *Zfy1* and *Zfy2* transcription in pachytene in the mutants compared to WT.

The current combination of multiple independent approaches provides a robust and internally consistent demonstration of impaired MSCI. RNA-FISH would be redundant to the broad array of complementary assays already included in the manuscript, and is highly unlikely to change the interpretation given the convergence of our cytological, molecular, and transcriptomic data. For these reasons, we did not add RNA-FISH to the revised manuscript.

Comment 7: • Line 308 - Although the data suggest that these mutants have no major defects in completing synapsis or progressing through meiotic prophase up to early pachytene stage, the authors should provide evidence that prophase staging is comparable across genotypes. Differences in staging or timing could otherwise confound the interpretation of the qRT-PCR results, since *Zfy1* and *Zfy2* are highly expressed at zygonema.

Response:

We thank the reviewer for drawing attention to this issue, which led us to realize that we inaccurately described the meiotic stages present in testes used for RT-qPCR experiments. Specifically, at 13 dpp, a major population, but not the absolute majority, of spermatocytes reaches early pachytene in the first meiotic wave, as shown for our standard C57BL/6J background in one of our previous studies (Papanikos et al. 2019; ref. [61]; DOI: 10.1016/j.molcel.2019.03.022). We have corrected the relevant description in the manuscript (lines 370-373) to state that “a major fraction” rather than “most” spermatocytes reach early pachytene at 13 dpp.

We agree that, in principle, if the mutants progressed through prophase with different dynamics than WT littermates, this could affect RT-qPCR results. However, extensive quantification of axis morphologies (Fig. 1e, ~3000 cells in both WT and *Sycp2*$^{e16/e16}$) did not show significant differences in the proportion of axis morphologies, strongly arguing against altered prophase progression. We note that whereas there are minor SC defects in the analyzed mutants, these are common phenotypes of recombination-defective mutants. These SC defects are not a result of altered prophase progression, as evidenced by the extensive quantification of IHO1 presence on unsynapsed axes (Supplementary Fig. 7d, over 1000 cells in each mutant). Because IHO1 specifically binds to unsynapsed axes, including the persistently unsynapsed XY axes and any aberrantly unsynapsed autosomes, its programmed disappearance serves as a robust marker to distinguish zygotene from early pachytene independently of SC formation. Using this criterion, our quantifications do not show significant differences in the ratio of late zygotene to early pachytene in WT, *Sycp2*$^{e16/e16}$, and *Hormad2*$^{-/-}$ mice. These observations are inconsistent with, and thus strongly

argue against, the hypothesis that meiotic progression is altered in the mutants in a manner that would significantly affect bulk transcript measurements.

Nevertheless, to further address the reviewer's concerns, we tested for potential differences in prophase stages in the exact same testes that were used for *Zfy1/2* expression assessment. To this end, we measured expression levels of early prophase markers (leptotene-zygotene enriched, see marker characteristics in Supplementary Fig. 11) using RT-qPCR in the RNA samples that were used for *Zfy1/2* analysis from the testes of WT and the *Sycp2*$^{e16/e16}$ mutant. These newly performed experiments are presented in Supplementary Fig. 13a, and show that autosomal marker genes that are enriched in zygotene are expressed at similar levels in WT and the *Sycp2*$^{e16/e16}$ mutant, arguing against the hypothesis that an increased proportion of zygotene cells explains the observed elevation of *Zfy1/2* transcript levels in the *Sycp2*$^{e16/e16}$ mutant. In short, the data strongly support the original conclusion that *Zfy1/2* silencing is inefficient in the *Sycp2*$^{e16/e16}$ mutant.

The revised text (lines 370-377) describing the RT-qPCR results is as follows:

"Using quantitative real-time PCR, we detected more than twofold higher expression of *Zfy1* and *Zfy2* in testes from *Sycp2*$^{e16/e16}$ compared with WT mice at 13 days postpartum (dpp) (Fig. 6d-e and Supplementary Fig. 13a), a developmental time point at which a major fraction of spermatocytes reaches early pachytene in the first meiotic wave in our C57BL/6J background[61]. In contrast, expression of autosomal marker genes enriched in leptotene and zygotene was comparable between WT and *Sycp2*$^{e16/e16}$ (Supplementary Fig. 13a), indicating that elevated *Zfy1* and *Zfy2* transcript levels in *Sycp2*$^{e16/e16}$ testes reflect reduced sex chromosome silencing rather than altered meiotic progression."

Comment 8: • Line 338 (Fig. 7A-B) - Even though it is common, there is a substantial variability in oocyte counts, especially for the WT and Sycp2 mutant mice. Please provide more details about how oocytes were quantified and discuss possible sources of variation.

Response:

Both ovaries from each female were serially sectioned throughout the entire ovary, and oocytes were detected by immunostaining for the nuclear oocyte marker NOBOX in every tenth section (8 µm sections). This systematic sampling interval was chosen to minimize the risk of counting the same oocyte twice while still comprehensively sampling the full ovary. This method allows reliable comparison between genotypes, as done in our previous studies. We have added clarifications to the Methods at lines 826-833 (paragraph starting with "To quantify oocyte numbers,…").

Regarding the observed variability, WT and *Sycp2*$^{e16/e16}$ animals from single-mutant crosses were pooled with WT and *Sycp2*$^{e16/e16}$ littermates derived from the double-mutant breeding schemes. Because we analyzed a large number of mice for these specific genotypes, the data inherently capture a broader range of natural biological variation across females. Furthermore, pooling mice from colonies that unavoidably had to be bred separately for single and double-mutant mice may contribute to the broad variation in oocyte numbers. We now point out these features of the samples in the revised legend for Figure 7 (lines 1613-1617). The revised part of the legend reads:

"WT and *Sycp2*$^{e16/e16}$ groups include animals from both single-mutant and double-mutant breeding schemes. The broad spread of oocyte numbers may partially reflect the pooling of mice from the separately bred, strain-specific colonies. Furthermore, the large number of analyzed mice, inherently reveals a wider range of natural variation across individuals.".

Comment 9: • Line 458-461 (Discussion) - The sentence "Together, our data indicate that SYCP2 CM–mediated recruitment of HORMAD2 to unsynapsed axes enables efficient ATR activation. Reinforced by positive feedback loops within the ATR-activating network, this mechanism sustains high chromatin-bound ATR activity, which ensures elimination of asynaptic meiocytes—a key safeguard against aneuploidy in mammalian meiosis." is inaccurate as written. In spermatocytes, this mechanism primarily enables silencing of the asynaptic XY chromosomal axes, not the elimination of the cells themselves. Therefore, the phrase "elimination of asynaptic meiocytes" is misleading in this context. I suggest replacing it with "silencing of asynaptic axes", or alternatively, if the authors wish to retain the concept of cell elimination, then "meiocytes" should be replaced with "oocytes", where ATR-dependent elimination indeed occurs.

Response:

We thank the reviewer for pointing out this inaccuracy. We agree that high chromatin-bound ATR activity does not ensure the elimination of asynaptic spermatocytes, but rather underlies the overall quality control mechanism of synapsis in both sexes. We have therefore rewritten the relevant paragraph (lines 548-552) to read:

"Together, our data indicate that SYCP2 CM–mediated recruitment of HORMAD2 to unsynapsed axes enables efficient ATR activation. Reinforced by positive feedback loops within the ATR-activating network, this mechanism sustains high chromatin-bound ATR activity, which promotes silencing of unsynapsed chromatin and underlies the surveillance and quality control of synapsis, thereby providing a key safeguard against aneuploidy in mammalian meiosis."

Comment 10: • Figure 1d and Sup Fig 3e) — H1t signal appears weaker in Supplementary Fig. 3e compared to Fig. 1d. Consider adjusting intensity for consistency.

Response:

We agree that the overall histone H1t signal appears weaker in Supplementary Fig. 3e compared with Fig. 1d. Both panels were acquired using matched exposure settings for the H1t channel and are displayed without panel-specific intensity adjustments. The two panels show different stages of the seminiferous epithelial cycle (Fig. 1d, stage IV; Supplementary Fig. 3e, stages V–VI). It is important to distinguish between H1t signal in pachytene spermatocytes versus post-meiotic spermatids, as both spermatogenic cell types can display histone H1t immunostaining. In Fig. 1d, the pachytene-associated H1t signal is lower than in the late pachytene cells shown in Supplementary Fig. 3e, whereas the spermatid-associated H1t signal appears slightly weaker in Supplementary Fig. 3e. Notably, H1t immunostaining in spermatids is stage-dependent, and H1t signal is lost as spermatids elongate at later epithelial stages. Thus, the apparent difference in spermatid-associated signal intensity between these panels likely reflects stage-dependent biological variation in the tubules shown, although regional variation in immunostaining efficiency across testis sections cannot be excluded. Regardless, to avoid misrepresenting relative signal intensities, we did not adjust H1t intensity separately between these panels.

Comment 11: • Figure 2d-f - HORMAD2 appears as a double band in some gels (2f) but not others (2d, e). Please clarify the source of this difference.

Response:

The reviewer is correct to note this variation. The observed variation is a technical consequence of the different gel running conditions used for these specific experiments, which we noted in the figure legend. Specifically, at the end of the legend for Figure 2 (lines 1508-1510), we stated: "Depending on SDS-PAGE conditions HORMAD2 is detected as a single (**d, e,** 15% PAGE) or double band (**f,** 4-16% gradient)." We hope this clarifies the source of the difference.

Comment 12: • Supplementary Table 1 – Presenting fertility as "pups per breeding week" is an unusual and not particularly informative metric. It would be more useful to report standard reproductive indicators, such as total pups per female, average litter size, and/or number of litters per female.

Response:

We have revised the fertility analysis to include the requested metrics. In the original manuscript, we used 'pups per breeding week' to allow for accurate comparisons of breeding performance

across the entire experimental period, because the total breeding duration varied among the different WT-mutant experimental pairs. In the revised manuscript, we have supplemented our original analysis (presented in Supplementary Table 1) with the requested indicators (total pups per female, average litter size, and number of litters per female) in a new Supplementary Table 2. To ensure standardized comparisons for these new metrics, we used the shortest breeding period as a common reference and recalculated the breeding performance for all mice based on this adjusted interval. These newly included data support our original conclusions.
